# The genomes of many yam species contain transcriptionally active endogenous geminiviral sequences that may be functionally expressed

**DOI:** 10.1093/ve/vev002

**Published:** 2015-05-26

**Authors:** Denis Filloux, Sasha Murrell, Maneerat Koohapitagtam, Michael Golden, Charlotte Julian, Serge Galzi, Marilyne Uzest, Marguerite Rodier-Goud, Angélique D’Hont, Marie Stephanie Vernerey, Paul Wilkin, Michel Peterschmitt, Stephan Winter, Ben Murrell, Darren P. Martin, Philippe Roumagnac

**Affiliations:** ^1^CIRAD-INRA-SupAgro, UMR BGPI, Campus International de Montferrier-Baillarguet, 34398 Montpellier Cedex-5, France; ^2^Computational Biology Group, Institute of Infectious Diseases and Molecular Medicine, University of Cape Town, Cape Town 4579, South Africa; ^3^Department of Integrative Structural and Computational Biology, The Scripps Research Institute, La Jolla, CA 92037, USA; ^4^Department of Pest Management, Faculty of Natural Resources, Prince of Songkla University, Hat Yai campus, Thailand 90120; ^5^CIRAD, UMR AGAP, TA A-108/03, Avenue Agropolis, F-34398 Montpellier Cedex 5, France; ^6^Royal Botanic Gardens, Kew, Richmond, Surrey, TW9 3AB, UK; ^7^DSMZ Plant Virus Department, Messeweg 11/12, 38102, Braunschweig, Germany; ^8^Department of Medicine, University of California, San Diego, La Jolla, CA

**Keywords:** endogenous viral sequences, geminivirus, yam, functional protein expression, selection analysis

## Abstract

Endogenous viral sequences are essentially ‘fossil records’ that can sometimes reveal the genomic features of long extinct virus species. Although numerous known instances exist of single-stranded DNA (ssDNA) genomes becoming stably integrated within the genomes of bacteria and animals, there remain very few examples of such integration events in plants. The best studied of these events are those which yielded the geminivirus-related DNA elements found within the nuclear genomes of various *Nicotiana* species. Although other ssDNA virus-like sequences are included within the draft genomes of various plant species, it is not entirely certain that these are not contaminants. The *Nicotiana* geminivirus-related DNA elements therefore remain the only definitively proven instances of endogenous plant ssDNA virus sequences. Here, we characterize two new classes of endogenous plant virus sequence that are also apparently derived from ancient geminiviruses in the genus *Begomovirus*. These two endogenous geminivirus-like elements (EGV1 and EGV2) are present in the *Dioscorea* spp. of the Enantiophyllum clade. We used fluorescence *in situ* hybridization to confirm that the EGV1 sequences are integrated in the *D. alata* genome and showed that one or two ancestral EGV sequences likely became integrated more than 1.4 million years ago during or before the diversification of the Asian and African Enantiophyllum *Dioscorea* spp. Unexpectedly, we found evidence of natural selection actively favouring the maintenance of EGV-expressed replication-associated protein (Rep) amino acid sequences, which clearly indicates that functional EGV Rep proteins were probably expressed for prolonged periods following endogenization. Further, the detection in *D. alata* of EGV gene transcripts, small 21–24 nt RNAs that are apparently derived from these transcripts, and expressed Rep proteins, provides evidence that some EGV genes are possibly still functionally expressed in at least some of the Enantiophyllum clade species.

## 1 Introduction

The geminiviruses (Family *Geminiviridae*) are a diverse group of viruses with circular single-stranded DNA (ssDNA) genomes composed of one or two components, varying in size from 2.5 to 3.0 kb, which are characteristically encapsidated within twinned incomplete icosohedral (or geminate) particles (Jeske 2009). All geminivirus genomes contain between four and eight genes and have a hairpin structure at their virion strand origins of replication (*v-ori*) that consists of a GC-rich stem and a loop containing a highly conserved AT-rich nonanucleotide motif (usually with the sequence TAATATTAC) (Jeske 2009). This hairpin is located within an intergenic region that also contains the bidirectional promoter elements and transcription start sites of diverging virion and complementary sense genes (Jeske 2009). The only two genes that are obviously conserved among all currently described geminiviruses are those encoding the coat protein (*cp*) and the replication associated protein (*rep*) (Bernardo et al. 2013). Although all known geminiviruses also express one or more proteins that are involved in virus movement, there is no detectable homology between the movement proteins of viruses in different genera (Jeske 2009).

Based on host ranges, vector specificities, genome organization, and genome-wide sequence similarities, the family *Geminiviridae* has been split into seven genera by the International Committee on the Taxonomy of Viruses: *Begomovirus, Curtovirus, Topocuvirus, Becurtovirus, Turncurtovirus, Eragrovirus*, and *Mastrevirus* (Adams, King, and Carstens 2013). However, recent discoveries of various highly divergent geminivirus-like ssDNA viruses that cannot reasonably be classified within these seven genera suggest that there likely exists far more diversity within this family than is currently represented by the established taxonomy (Krenz et al. 2012; Loconsole et al. 2012; Bernardo et al. 2013). Although the begomoviruses, curtoviruses, and topocuviruses share relatively similar genome structures and are known to naturally infect only dicotyledonous plants, viruses in the other genera have a variety of unique genomic features and have been found to infect both monocotyledonous and dicotyledonous plants (Yazdi, Heydarnejad, and Massumi 2008; Varsani et al. 2009a; Krenz et al. 2012; Loconsole et al. 2012; Bernardo et al. 2013).

Geminiviruses replicate via both rolling circle and recombination-dependent mechanisms (Jeske, Lutgemeier, and Preiss 2001). Although genetic recombination between different geminivirus genomes coinfecting the same cells frequently occurs during the replication process, the discovery of geminivirus sequences integrated into the genomes of various plant species indicates that recombination with host nuclear DNA may also be more common than previously thought (Kenton et al. 1995; Bejarano et al. 1996; Ashby et al. 1997; Murad et al. 2004). Although so-called endogenous geminivirus sequences are clearly apparent within the recently published genome sequences of *Lactuca sativa* (common lettuce, GenBank accession: PRJNA68025), *Malus domestica* (common apple tree, GenBank accession: PRJNA28845), *Coffea canephora* (common coffee, GenBank accessions: CDP12517, CDP12572, CDP16111, CDP16477, CDP18020, CDP18767, CDP19427, CDP21894, CDP18021, CDP18667, CDP20185, and CDP20985), and *Populus trichocarpa* (black cottonwood tree, GenBank accession: PRJNA17973) (Liu et al. 2011; Martin et al. 2011), the best studied of these integrated geminivirus sequences are the so-called ‘geminivirus-related DNA’ (GRD) elements within the genomes of various *Nicotiana* species (Kenton et al. 1995; Bejarano et al. 1996; Ashby et al. 1997; Murad et al. 2004).

GRD elements have been classified into three groups: GRD2, GRD3, and GRD5. GRD2 is represented by five to fifteen copies on chromosome 4 of *N.tomentosa*. The other GRD elements are found as three related repeat classes, GRD5, GRD53, and GRD3, clustered as multiple direct repeats on homologous group 4 chromosomes (GRD5 and GRD53) or on chromosome 2 (GRD3) in several *Nicotiana* species. Each GRD element contains a degenerate and truncated begomoviral *rep* gene and an intergenic region fragment carrying a geminivirus virion-sense origin of replication (v-*ori*) -like sequence (Kenton et al. 1995; Bejarano et al. 1996; Ashby et al. 1997; Murad et al. 2004).

Endogenous viral sequences such as the GRD elements are essentially ‘fossil records’ that have the potential to reveal the genomic features of long extinct virus species (Katzourakis 2013). Upon integration, such sequences, if non-functional, would have begun evolving under neutral genetic drift at approximately 10^–9^ substitutions per site per year (Huang et al. 2012), respectively, approximately 10 (Lefeuvre et al. 2011) to 10,000 (Duffy and Holmes 2008) times slower than the long- and short-term substitution rates of ‘free living’ geminiviruses. Given the approximate timing of such integration events, the comparison of endogenous sequences with their distant contemporary relatives can be used to both determine long-term viral genomic substitution rates (Gilbert et al. 2009; Gilbert and Feschotte 2010; Lefeuvre et al. 2011) and to date events in the deep evolutionary history of viruses (Emerman and Malik 2010; Katzourakis and Gifford 2010).

Analyses of the multiple geminivirus *rep* fragments within the genomes of various *Nicotiana* species have indicated that they have likely been evolving under neutral genetic drift (Murad et al. 2004) and that the GRD5 and GRD3 elements likely became integrated approximately 3 million years ago (MYA) and 0.2 MYA, respectively (Gibbs et al. 2010; Lefeuvre et al. 2011). These integration times suggest that long-term begomovirus substitution rates (i.e. over hundreds of thousands of years) are orders of magnitude lower than their short-term substitution rates (i.e. those occurring over tens of years (Duffy and Holmes 2008; Gibbs et al. 2010)), such that (1) the most recent common geminivirus ancestor may have existed 50 or more MYA (Gibbs et al. 2010; Lefeuvre et al. 2011) and (2) the split between the Old-World and the New-World begomovirus lineages may have been directly attributable to a dramatic global cooling event 35 MYA, which effectively closed the Bering land bridge between Asia and North America as a dispersal route for tropical/temperate plant and animal species (Lefeuvre et al. 2011). The power of such analyses has, however, been hampered by the fact that they have relied on only a single set of closely related integrated sequences. For example, in a study by Lefeuvre et al. (2011), this resulted in extremely broad credibility intervals, ranging from 2 to 80 MYA, for the estimated date of the split between the Old and New World begomovirus lineages. The discovery and analysis of additional fossilized geminivirus sequences within the genomes of other plant species would certainly help to more accurately date events deep in the evolutionary history of this family: Events such as the most recent geminivirus common ancestor and the origins of the various geminivirus genera.

Here, we characterize two new endogenous geminivirus elements (EGV1 and EGV2), which we initially discovered within the genome of *Dioscorea alata* (water yam). We also show the presence of closely related sequences of EGV1 or EGV2 in twenty-two other *Dioscorea* species (all closely related to *D.* *alata* in the Enantiophyllum clade). We demonstrate that, unlike the GRD elements found in *Nicotiana*, some of the *rep* gene lineages within these sequences display post-integration signals of purifying selection. Finally, we provide evidence that some EGV genes are possibly still functionally expressed in various yam species.

## 2 Materials and methods

### 2.1 Inventory of viral sequences within *D.* *alata*

Attempts were made to make an inventory of virus species infecting water yam (*D.* *alata*) accessions held at the CIRAD yam quarantine station in Montpelier, France. The virus identification strategy employed two approaches: (1) virion-associated RNA isolation, reverse transcription and sequencing and (2) *in silico* screening of publically available expressed sequence tag data (EST) from yam.

### 2.2 Virion-associated RNA isolation, reverse transcription, and sequencing

Partial purification of potential viral particles from *D.* *alata* acc. 313 leaf samples was performed as described previously (Jones et al. 2001). Pellets were resuspended in 150 µl of 1X RQ1 DNase buffer (Promega), which was then treated with 15 U of RQ1 DNase (Promega) and 10.5 U of RNase A (Qiagen) at 37°C for 2 h to digest non-particle-protected nucleic acids. RNA was extracted with an RNeasy Plant Mini Kit (Qiagen). Random RT-polymerase chain reaction (PCR) amplification was then performed with the TransPlex® Whole Transcriptome Amplification (Sigma-Aldrich) kit according to the manufacturer’s protocol. Potential RNA and DNA virus genome amplicons ranging in size from 200 to 1,000 bp were gel purified (SV Gel and PCR Clean-Up System (Promega)) and inserted into pGEM®-T Easy vector as recommended by the manufacturer (Promega). The inserts were amplified by PCR using the universal primers T7 and SP6, and fragments >250 bp were sequenced by single-pass double-stranded analysis (Cogenics) using the same primers. Sequence similarity searches were performed using BlastN and BlastX methods (Altschul et al. 1990).

### 2.3 *In silico* screening of publically available EST from yam

A systematic search of assembled sequences (using the CAP3 sequence assembly program (Huang and Madan 1999) from publicly available *D.* *alata* EST resources (GenBank accession numbers: HO809681–HO825421, HO825422–HO840419, and HO850622–HO864016) was performed using the BlastN and BlastX methods implemented in the software KoriBlast 3.1 (KoriLog) with a maximum *E*-value threshold of 10^−^^4^.

### 2.4 Isolation of EGV1 and EGV2 flanking regions and whole EGV1 and EGV2 *rep* genes

Given that the virion-associated RNA isolation method includes a DNAse step, it was somewhat unexpected that a geminivirus-like sequence (designated EGV1) was discovered. Another distinct geminivirus-like sequence (designated EGV2) was also detected with the *D.* *alata* EST screening procedures (EGV2).

Two pairs of outward facing primers (i.e. primers with orientations directed away from one another rather than towards one another as is the case with standard PCR) were designed for recovering the full-length EGV1 and EGV2 sequences (Supplementary Table S1) from *D.* *alata* acc. 313 and *D.* *nummularia* acc. 206. We used these primers to test for the presence of tandemly repeated EGV1 and EGV2 sequences in both *Dioscorea* species. We included *D.* *nummularia* in these experiments to determine whether tandemly repeated EGV sequences were found in multiple different *Dioscorea* species. No amplification products were obtained using the EGV2 outward facing primers. Inverse PCR was then performed as described by Ochman, Gerber, and Hartl (1988) to recover flanking regions of EGV2 from *D.* *nummularia* acc. 206, *D.* *persimilis* acc. 271, and *D.* *alata* acc. 313.

Based on the alignment of the full-length 2.6-kb EGV1 sequence and approximately 2.9 kb EGV2 fragments from *D.* *alata, D.* *persimilis,* and *D.* *nummularia* (produced using ClustalW with default settings (Larkin et al. 2007)), three pairs of primers were designed for amplifying whole EGV1 and EGV2 *rep* genes. One pair was designed for amplifying the *D.* *alata* EGV1 *rep* gene, another for the *D.* *nummularia* EGV1 *rep* gene, and a third, ‘universal’ primer pair, for amplifying EGV2 *rep* genes from *D.* *nummularia* and *D.* *alata* (Supplementary Table S1). The fifteen Asian yam species belonging to the Enantiophyllum clade listed in Table 1 were PCR tested with the three sets of primers. Amplification reactions were carried out in a 25 μl volume containing 20 ng of plant genomic DNA, 0.4 µM of each primer (Supplementary Table S1), and GoTaq Hot Start Master Mix (Promega) following the manufacturer’s protocol. The amplification conditions consisted of an initial denaturation at 95 °C for 2 min, followed by thirty-five cycles of denaturation at 94 °C for 1 min, annealing at 55°C for 1 min, extension at 72°C for 90 s, and finally an extension step of 72°C for 10 min. Amplified DNA fragments were gel purified (using the SV Gel and PCR Clean-Up System from Promega) and inserted into pGEM®-T Easy vector (Promega) following the manufacturer’s protocol. The universal T7 and SP6 primers were used for sequencing one clone from each of the *Dioscorea* species that PCR-tested positive except for *D.* *transversa* acc. 336 and *D.* *persimilis* acc. 271, for which eleven and eight clones were sequenced, respectively. Sequence data of all the amplified fragments was obtained by single-pass double-stranded analysis (Beckman Coulter Genomics).

**Table 1.** PCR detection of EGV1 and EGV2 sequences in the genomes of a collection of yam species.

Species	No.	Section	Clade	Country	Origin of the species	EGV1 sequence	EGV2 sequence
*D. wallichii* Hook.f.	14472	Enantiophyllum	Enantiophyllum	Thailand	South East Asia	+	+
*D. inopinata* Prain & Burkill	15674	Enantiophyllum	Enantiophyllum	Thailand	South East Asia	+	+
*D. oryzetorum* Prain & Burkill	15671	Enantiophyllum	Enantiophyllum	Thailand	South East Asia	+	+
*D. alata* L.	313	Enantiophyllum	Enantiophyllum	India	South East Asia	+	+
*D. persimilis* Prain & Burkill^a^	271	Enantiophyllum	Enantiophyllum	Vietnam	South East Asia	+	+
*D. nummularia* Lam.	206	Enantiophyllum	Enantiophyllum	Vanuatu	Melanesia	+	+
**D**. *transversa* R.Br.	336	Enantiophyllum	Enantiophyllum	Vanuatu	Melanesia	+	+
*D. glabra* Roxb*.*	21051	Enantiophyllum	Enantiophyllum	Thailand	South East Asia	+	+
*D. calcicola* Prain & Burkill	6215	Enantiophyllum	Enantiophyllum	Thailand	South East Asia	+	+
*D. hamiltonii* Hook.f.^a^	6210	Enantiophyllum	Enantiophyllum	Thailand	South East Asia	+	+
*D. brevipetiolata* Prain & Burkill	14475	Enantiophyllum	Enantiophyllum	Thailand	South East Asia	+	+
**D**. *opposita* Thunb.	265	Enantiophyllum	Enantiophyllum	France	China	+	+
*D. decipiens* Hook.f.	6481	Enantiophyllum	Enantiophyllum	Thailand	South East Asia	+	+
*D. cirrhosa* Lour.	15672	Enantiophyllum	Enantiophyllum	Thailand	South East Asia	+	+
*D. lanata* Bail	6181	Enantiophyllum	Enantiophyllum	Socotra	Arabian Peninsula	−	−
**D.*** abyssinica* Hochst. ex Kunth	109	Enantiophyllum	Enantiophyllum	Benin	West Africa	+	+
*D. cayenensis* Lam.	1	Enantiophyllum	Enantiophyllum	Haïti	West Africa	+	+
*D. praehensilis* Benth.	255	Enantiophyllum	Enantiophyllum	Benin	West Africa	+	+
*D. rotundata* Poir.	389	Enantiophyllum	Enantiophyllum	Benin	West Africa	+	+
*D. schimperiana* Hochst. ex Kunth	21044	Enantiophyllum	Enantiophyllum	Malawi	East Africa	+	−
*D. schimperiana* Hochst. ex Kunth	22295	Enantiophyllum	Enantiophyllum	Malawi	East Africa	+	−
**D.*** togoensis* R.Knuth	114	Enantiophyllum	Enantiophyllum	Guinea	West Africa	−	+
*D. minutiflora* Engl.	031	Enantiophyllum	Enantiophyllum	Madagascar	West Africa	+	+
*D. bulbifera* L.	272	Opsophyton	Compound leafed	Papua New Guinea	South East Asia	−	−
**D.** *dumetorum* (Kunth) Pax	67	Lasiophyton	Compound Leafed	Burkina Faso	West Africa	−	−
*D. pentaphylla* L.	Dp1038	Lasiophyton	Compound-Leafed	?	?	−	−
**D.*** maciba* Jum. & H.Perrier	14348	?	Malagasy	Madagascar	Madagascar	−	−
*D. sansibarensis* Pax	269	Macroura	Malagasy	Benin	West Africa	−	−
*D. birmanica* Prain & Burkill	15677	Stenophora	Birmanica	Thailand	South East Asia	−	−
**D.*** esculenta* (Lour.) Burkill	002	Combilium	Birmanica	Madagascar	South East Asia	−	−
*D. buchananii* Benth.	15073	Rhacodophyllum	Africa	Zambia	Southern Africa	−	−
**D.** *elephantipes* (L'Hér.) Engl.	328	Testudinaria	Africa	France	Southern Africa	−	−
**D.** *communis* (L.) Caddick & Wilkin	310	?	Europe	France	Europe	−	−
**D.** *amaranthoides* C.Presl	16523	Strutantha	New World	Bolivia	South America	−	−
*D. galeottiana* Kunth	6204	?	New World	Mexico	North America	−	−
*D. membranacea Pierre ex Prain & Burkill*	21050	?	Stenophora	Thailand	South East Asia	−	−
*D. balcanica* Koanin	266	Stenophora	Stenophora	France	Europe	−	−
*D. villosa* L.	267	Stenophora	Stenophora	France	North America	−	−
*D. trifida* L.f.	78	Macrogynodium	Macrogynodium	French Guyana	South America	−	−
**D.** *melastomatifolia* Uline ex Prain	368	?	?	French Guyana	South America	−	−
*D. pubescens* Poir.	367	?	?	French Guyana	South America	−	−

^a^Although *D. persimilis* and *D. hamiltonii* are considered to be the same species, from the perspective of their choroplast genomes they are genetically distinct. They were therefore treated as separate species here.

### *2.5 *Fluorescence *in situ* hybridization

We then tested the hypothesis that the geminivirus-like sequences were integrated within the *D.* *alata* genome, using fluorescence *in situ* hybridization (FISH). Root tips of the diploid *D.* *alata* plants acc. 256 (2 *n* = 40) were collected on bright and sunny mornings (to ensure that cells were actively dividing). Chromosome preparations and hybridization were performed as described previously (Chabannes et al. 2013). The plant-derived geminivirus-like sequence EGV1 (2.6 kb) obtained from *D.* *alata* acc. 313 was used as a probe. A 45 S rDNA probe was used as a control.

### 2.6 Search of circular viral genomes

Attempts were made to detect the presence of small episomal EGV-derived DNA sequences (i.e. geminivirus-like replicative forms) within *Dioscorea* using rolling circle amplification (RCA) and sequencing. DNA was extracted from *D.* *alata* acc. 313 and *D.* *transversa* acc. 336 leaf samples with a DNeasy Plant Mini Kit (Qiagen), and potential circular viral genomes located within these samples were subjected to sequence-independent RCA using Phi29 DNA polymerase using a cocktail of random and specific primers (YLCV2F and YLCV1R) used for detecting EGV1 (Supplementary Table S1) as described previously (Inoue-Nagata et al. 2004). Positive control DNA (pUC19) was also used. Two restriction enzymes, namely *Acu*I and *Dra*I, which were, respectively, expected to cut twice and once in EGV1 were used to identify episomal EGV1 sequences.

### 2.7 Characterization of multiple EGV1 repeats from *D. alata*

Two pairs of primers, one located within the replication enhancer gene (*ren*) called the ‘*ren-ren*’ primer pair and one located within the *rep* gene called the ‘*rep-rep*’ primer pair (Supplementary Table S1), were designed for amplifying two small fragments (572 and 444 bp) and potentially two longer fragments (3,214 and 3,086 bp) that would encompass the small fragment and include one partial copy of EGV1. *D.* *alata* acc. 313 plant was PCR tested with these two sets of primers. Amplification conditions described above were modified to obtain multiple EGV1 copies from each plant: reactions were carried out in 50 μl volumes and contained 20 ng of plant genomic DNA, 0.2 µM of each primer (Supplementary Table S1) and GoTaq Hot Start Master Mix (Promega) following the manufacturer’s protocol. The following amplification conditions were used: initial denaturation (95°C for 2 min), followed by thirty-five cycles of denaturation (94°C for 10 s), annealing (55°C for 30 s) and extension (68°C for 10 min), and a final extension step (68°C for 10 min). Fragments of 3.2 kb amplified with long range ‘*ren-ren*’ and ‘*rep-rep*’ primer pairs were gel purified (SV Gel and PCR Clean-Up System from Promega) and ligated into pGEM-T Easy vector (Promega). Ten and seven recombinant clones were obtained from ‘*ren-ren*’ and ‘*rep-rep*’ amplification products, respectively. These seventeen clones were sequenced by single-pass double-stranded analysis (Beckman Coulter Genomics) using a primer walking approach. Primer sequences were trimmed from the final analysed sequences yielding twenty-four partial *rep* sequences (each 402-bp long) that were then aligned using ClustalW with default settings (Larkin et al. 2007).

### 2.8 Distribution of geminivirus-like sequences within the genomes of *Dioscoreacea* species

DNA was extracted from a further forty yam species and screened for the presence of EGV1 and EGV2 using four broad spectrum primer pairs (Supplementary Table S1), designed according to geminivirus-like sequences found in an unpublished *D.* *alata* and *D.* *rotundata* EST database produced by the Agropolis Resource Centre for Crop Conservation, Adaptation and Diversity (Montpellier, France).

### 2.9 Testing for the seed transmissibility of the EGV1 and EGV2 sequences

Nine *D.* *alata* plants collected worldwide were also tested for the presence of EGV1 and EGV2 using two primer pairs (EGV1_ Detection_2F/EGV1_Detection_2F and EGV2_Detection_2F/EGV2_Detection_2R; Supplementary Table S1). These *D.* *alata* plants included two grown from seeds under virus-free conditions (Supplementary Table S2). The purpose of this experiment was to test whether the detected geminivirus sequences were seed transmitted; to our knowledge, there is presently no evidence of any non-endogenous geminiviruses being seed transmissible. Amplification reactions were carried out in 25 μl volumes and contained 20 ng of plant genomic DNA, 0.2 µM of each primer (Supplementary Table S1) and GoTaq Hot Start Master Mix (Promega) following the manufacturer’s protocol. The amplification conditions used were initial denaturation (95°C for 2 min), followed by thirty-five cycles of denaturation (94°C for 1 min), annealing (55°C for 1 min) and extension (72°C for 70 s), followed by a final extension step (72°C for 10 min).

### 2.10 Cloning and sequencing of DNA fragments

Amplified DNA fragments obtained from PCR assays (EGV1 and EGV2 *rep* genes, *rbc*L and *mat*K partial genes—see below) were gel purified (using the SV Gel and PCR Clean-Up System from Promega) and inserted into pGEM®-T Easy vector (Promega) following the manufacturer’s protocol. The universal T7 and SP6 primers were used for sequencing. Sequence data of all the amplified fragments were obtained by single-pass double-stranded analysis (Beckman Coulter Genomics) using a primer walking approach when needed and was further assembled using DNAMAN for windows (Lynnon Corporation).

### 2.11 siRNA extraction and sequencing

Small RNA, including small interfering RNA (siRNA), was prepared from *D.* *alata* using RNAzol B (WAK Chemie, Germany) essentially following the manufacturers protocol (RNAzol®RT Brochure, 2010; Molecular Research Center, Inc. Cincinnati, OH). After quality control, small RNA preparations were used for library preparation and subjected to high-throughput sequencing on an Illumina ‘Hi-Seq 2000’ instrument using the services of a commercial company (Fasteris SA, Plan-les-Ouates, Switzerland). Bioinformatic analyses of viral siRNAs were performed as recently described (Seguin et al. 2014).

### 2.12 Western blotting analysis

One peptide, LEGRAQVTNNRFDL, putatively encoded by EGV1 *rep* gene from *D.* *alata* acc. 313 was selected because it did not display significant similarity (all obtained BlastX or tBlastN, *E-*values > 10^−^^1^) to any Dioscoreales (NCBI Blast Dioscoreales taxid: 40548) proteins from the non-redundant protein sequence (nr), the metagenomic protein (env_nr), and the transcriptome shotgun assembly protein (tsa_nr) NCBI databases. However, BlastX comparisons between LEGRAQVTNNRFDL and all geminiviral sequences deposited in GenBank (NCBI Blast Geminiviridae taxid: 10811) indicated that the highest identity score was detected with the *Sida micrantha mosaic virus* Rep protein (accession number CAD89704.1, highest percent identity = 80%, *E* value = 1.10^−^^4^). In addition, *Tomato yellow leaf curl virus*-Mld (TYLCV-Mld, accession number AJ865337) shared 47 per cent identity with the selected EGV1 peptide. Antibodies were obtained using the ‘Rabbit Speedy mini’ approach developed by Eurogentec S.A., Belgium; 0.35 g of leaf material was homogenized on ice in 1.5 ml of tris buffer saline (TBS; 0.9% NaCl, Tris 50 mM KCl, pH 7.4). Samples were then centrifuged for 5 min at 6,000 g. Supernatants were mixed with a 3:1 ratio in Laemmli 2X loading buffer (4% sodium dodecyl sulphate, 4% 2-mercaptoethanol, 30% glycerol, 100 mM Tris pH 6.8, bromophenol blue) and were further denaturated by heating at 105°C for 5 min prior to 12 per cent PAGE under denaturating conditions (10 µl/sample). Separated proteins were then transferred onto a nitrocellulose membrane using a semi-dry apparatus (CBS) according to the manufacturer’s instructions. Membranes blocked in TBST buffer (TBS with 0.1% tween 20) supplemented with 5 per cent skimmed milk were incubated with primary antibodies for 2 h at room temperature in the same buffer (dilution 1:1,000 of the rabbit polyclonal antibodies raised against the peptide LEGRAQVTNNRFDL). After three rinses in TBST buffer, the membranes were incubated in phosphatase-alkaline anti-rabbit IgG (Santa Cruz Biotechnology) for 1 h at room temperature in TBST buffer (dilution 1:1,500). The presence of the Rep protein was detected after rinses by using the nitro blue tetrazolium (NBT)/5-bromo-4-chloro-3-indolyl phosphate (BCIP) colorimetric reaction. A turnip plant infected by Cauliflower mosaic virus and a tomato plant infected by TYLCV-Mld were used as negative controls.

### 2.13 Reconstruction of the yam species phylogeny

The plastid genes, *rbc*L and *mat*K, were amplified as described previously (Wilkin et al. 2005) from all Asian and African yam species belonging to the Enantiophyllum clade that were absent from the yam species phylogeny published by Wilkin *et al*. (2005): These included *D.* *alata*, *D.* *persimilis*, *D.* *nummularia*, *D.* *transversa*, *D.* *calcicola*, *D.* *hamiltonii*, *D.* *opposita*, *D.* *cirrhosa*, *D.* *abyssinica*, *D.* *cayenensis*, *D.* *praehensilis*, *D.* *rotundata*, *D.* *minutiflora,* and *D.* *togoensis*. Concatenated *rbc*L and *mat*K sequences were aligned using the CLUSTALW method implemented in MEGA (with default settings, alignment file is provided as Supplementary Material). Maximum likelihood phylogenetic trees were constructed using PHYML3 (Guindon et al. 2010) with a TN93+G4 nucleotide substitution model (selected as best fit by RDP4.23 (Martin et al. 2010)) and 1,000 bootstrap replicates used to test the support of branches.

### 2.14 Reconstruction of the yam-derived EGV1 and EGV2 Rep phylogenies

Thirty-seven EGV1 and twenty EGV2 replication-associated protein gene (*rep*) nucleotide sequences collectively derived from fourteen different yam species were aligned using the MUSCLE method (Edgar 2004) implemented in MEGA (Tamura et al. 2011) (with default settings, alignment file is provided as Supplementary Material). Maximum likelihood phylogenetic trees were constructed using PHYML3 (Guindon et al. 2010) (with 100 full bootstrap replicates using both the nearest neighbour interchange and subtree prune and regraft search strategies) with automated best-fit model selection under the Akaike information criterion carried out using RDP4.23 (Martin et al. 2010). RDP4.23 was additionally used (with default settings) to detect evidence of recombination (or gene conversion) between EGV1 and EGV2 *rep* sequences.

### 2.15 Phylogenetic analysis of geminivirus *rep* and *ren* coding regions

We focused on the *rep* and *ren* coding regions to determine the evolutionary relationships of EGV1 and EGV2 to the various major geminivirus lineages. We assembled datasets consisting of sixty-nine Rep amino acid sequences representing the entire breadth of known geminivirus diversity. Besides the inferred EGV1 and EGV2 Rep amino acid sequences from *D.* *alata*, the Rep dataset contained twenty-four Mastrevirus sequences, eighteen Begomovirus sequences, eight Curtovirus sequences, two Becurtovirus sequences, one Eragrovirus sequence, one Turncurtovirus sequence, one Topocuvirus sequence, and one sequence each from the divergent geminivirus-like ssDNA viruses recently discovered infecting citrus plants (Citrus chlorotic dwarf-associated virus, CCDaV, genome accession: JQ920490), grapevines (Grapevine Cabernet Franc-associated virus, GCFaV, genome accession: JQ901105), *Euphorbia caput-medusae* (Euphorbia caput-medusae latent virus, EcmLV, genome accession: HF921459), and French bean (French bean severe leaf curl virus, FbSLCV, genome accession: NC_018453). In addition to these sequences, the Rep dataset contained translated amino acid sequences representative of geminivirus-like Rep sequences that might be expressed from integrated geminivius-like sequences that are potentially found within various plant genomes: *L. sativa* (common lettuce, GenBank accession: PRJNA68025), *M. domestica* (common apple tree, GenBank accession: PRJNA28845), *P. trichocarpa* (black cottonwood tree, GenBank accession: PRJNA17973), *Fraxinus angustifolia* (narrow-leafed ash, GenBank accession: AY760062), *Camellia sinensis* (tea plant, GenBank accession: HP764465), and *Bituminaria bituminosa* (Arabian pea, GenBank accession: JL856919). In addition, two divergent Rep sequences from ssDNA replicons closely related to geminiviruses were used to root the phylogeny: one derived from the witches’ broom-associated phytoplasmal plasmid and the other from the geminivirus-like mycovirus *Sclerotinia sclerotiorum* hypovirulence-associated DNA virus (SsHADV, genome accession: NC_013116). Because of the extremely distant relationships that existed even between the inferred amino acid sequences of these proteins, it was not possible to accurately align the sequences. We therefore accounted for alignment uncertainty using a Bayesian approach to simultaneously estimate phylogenetic trees and alignments with the computer program BAli-Phy version 2.2.1 (Suchard and Redelings 2006) (alignment file is provided as Supplementary Material). Default BAliBali-Phy settings were used with the LG2008 + gwF (Le and Gascuel 2008) amino acid substitution model (previously determined to be the best fit amino acid substitution model for these sequences (Varsani et al. 2009b)) in conjunction with the RS07 (Suchard and Redelings 2006) insertion-deletion model. Convergence was checked by running two independent chains for each set of sequences. The estimated sample size was calculated using the program Tracer (http://tree.bio.ed.ac.uk/software/tracer/) and the combined estimated sample size in each case was greater than 200.

The Ren sequence dataset contained, in addition to the forty-seven Ren sequences from *D.* *inopinata, D.* *oryzetorum, D.* *alata, D.* *persimilis, D.* *nummularia, D.* *transversa, D.* *glabra, D.* *calcicola, D.* *hamiltonii, D.* *brevipetiolata, D.* *opposita, D.* *decipiens, D.* *cirrhosa, D.* *lanata,* and *D.* *schimperiana*, fifteen begomovirus, one curtovirus, and one topocuvirus sequences. These sequences were aligned using MUSCLE method implemented in MEGA (with default settings, alignment file is provided as Supplementary Material) and were used to construct a maximum likelihood tree with PhyML3 using a JTT amino acid substitution model with branch supports being tested with 100 bootstrap replicates.

### 2.16 Selection analyses

To investigate the nature of the selective pressure across the combined EGV1 and EGV2 phylogenies, we implemented a codon model (Muse and Gaut 1994) of episodic selection (Kosakovsky Pond et al. 2011; Murrell et al. 2012a,b). Lineages on the phylogeny were partitioned into three distinct groups: EGV1, EGV2, and the connecting lineage which we expected to be evolving under evolutionary pressures typical for circulating viruses if EGV1 and EGV2 sequences had been independently endogenized. Within each partition, we let *ω* (*dN/dS*) along each branch for each site be randomly drawn from one of three categories: *ω*_1_ < 1, *ω*_2_ = 1 and *ω*_3_ > 1. The proportions of *ω*_1_ and *ω*_2_ are governed by a set of two partition-specific parameters, *p*_1_ and *p*_2_, and 1 − (*p*_1_ + *p*_2_) is the proportion of *ω*_3_. Each partition thus had five parameters. This branch-wise and site-wise ‘random effect’ independence is achieved through the same process-mixture approach used in bsREL (Kosakovsky Pond et al. 2011) and MEME (Murrell et al. 2012b). In fact, our model can be seen as a simplification of bsREL, which allows each branch to have three independently estimated *ω* categories. Here, by sharing parameters—and thus pooling the evidence—from all branches within an entire partition (where we have an *a priori* justification that they should share a selection profile), we gained power to detect more subtle effects than those that might be detectable along single branches. This aspect of the selection analysis approach that we adopted was particularly relevant because of the low degrees of nucleotide divergence among the EGV1 and EGV2 sequences. Branch lengths, and a general reversible nucleotide substitution model, were parameters shared by all three partitions, and equilibrium frequencies were modelled using the corrected CF3x4 estimator (Kosakovsky Pond et al. 2010).

Although our full model supported three distinct *ω* categories for each partition, we introduced constraints to test for purifying selection within a particular partition. For example, when testing for purifying selection within the EGV1 integration partition, we constructed both a null model with only neutral evolution (*ω*_2_ = 1) on that partition and an alternative model that allowed neutral and purifying selection (respectively, *ω*_2_ = 1 and *ω*_1_ < 1). As the alternative model reduces to the null model and has two additional parameters, we obtained *P* values from a likelihood ratio test using a *χ*^2^ distribution with two degrees of freedom (Self and Liang 1987), which we should expect to be conservative (Zhang, Nielsen, and Yang 2005). When testing for purifying selection within any partition, the parameters on the other two background partitions (five on each) remained unconstrained, so as not to bias the foreground partition tests. We also constructed an analogous test for positive selection (against a null model that allowed purifying and neutral selection), but omit it from discussion here because we found no evidence of positive selection in any of the three EGV1 and EGV2 partitions.

The sequence datasets analysed by these methods included fifteen EGV1 and fourteen EGV2 Rep encoding sequences that contained no premature stop codons, frameshift mutations or evidence of recombination (tested with RDP4.23;(Martin et al. 2010), alignment file is provided as Supplementary Material).

## 3 Results and discussion

### 3.1 Inventory of viruses in *D.* *alata*

Nine distinct virus-like sequences were detected through screening of the publically available *D.* *alata* EST database (dbEST Id: 71472229). In addition, one virus-like sequence was recovered using routine viral diseases diagnostic procedures (Supplementary Table S3). These sequences displayed significant similarity (BlastN/BlastX, *E-*value < 10^−^^5^) to members of the *Secoviridae* family (three sequences similar to viruses in the genus *Sadwavirus* and three sequences that cannot be classified within genera of the *Secoviridae* family)*, Geminiviridae* (two sequences designated as EGV1 (209 bp) and EGV2 (316 bp) similar to viruses in the genus *Begomovirus*), *Potyviridae* (one sequence), and *Caulimoviridae* (one sequence).

### 3.2 Characteristics of the EGV1 and EGV2 sequences

We first used outward facing primers to determine the sequences of complete EGV1 and EGV2 elements. This approach was successful for assembling one 2,642-bp and one 2,136-bp EGV1 fragment from *D. alata *and *D.* *nummularia*, respectively. Unexpectedly, no amplification product was obtained for EGV2. However, an inverse PCR approach enabled the assembly of 2,913, 2,920, and 2,937 bp EGV2 fragments from *D. alata, D.* *persimilis,* and *D.* *nummularia*, respectively.

Both the 2,642-bp EGV1 unit obtained from *D. alata* and the 2,136-bp EGV1 unit obtained from *D.* *nummularia* contain a GC-rich sequence resembling the conserved hairpin structures found at geminivirus virion strand origins of replication (*V-oris*), including a characteristic TAATATTAC sequence in the putative loop region (Supplementary Fig. S1). Potential TATA box sequences in what would be the complementary sense gene promoter of a geminivirus genome (located at position 2586) were also present in both sequences. Also, the predicted proteins expressed by these sequences had detectable homology to begomovirus replication enhancer (Ren; BlastX: closest hit to Tomato leaf curl Joydebpur virus, length: 134 aa, maximum % identity = 57%, *E*-value = 9.10^−45^ for the *D. alata* sequence) and replication-associated protein sequences (Rep; BlastX: closest hit to Okra mottle virus, length: 134 aa, maximum % identity = 59%, *E*-value = 5.10^−154^ for the *D. alata* sequence). We were, however, unable to detect homologues of either coat protein or transcription activator protein genes in the *D. alata* and *D.* *nummularia* EGV1 sequences, despite these genes being found in almost all known begomoviruses.

The *rep*- and *ren*-like EGV1 sequences from both yam species were very similar (respectively, sharing 93.6% and 98.7% identity), but the 3’ portion of the *D.* *nummularia ren*-like sequence was approximately 150 nt shorter than that of *D. alata* (which was approximately the same length as *ren* sequences found in ‘free-living’ geminiviruses).

The EGV2 unit lacked a detectable *v-ori* homologue, but the predicted proteins potentially expressed by the 2,936 bp *D. alata* EGV2 sequence included both a Rep homologue (BlastX: closest hit to Macroptilium yellow spot virus, length: 348 aa, maximum % identity = 59%, *E*-value = 2.10^−142^) and a truncated Ren homologue (BlastX: closest hit to Clerodendrum golden mosaic China virus, length: 57 aa, maximum % identity = 72%, *E*-value = 1.10^−20^) that is missing an approximately 100 nt long region in the central part of the gene. As with the EGV1 sequences, the EGV2 sequence was missing a detectable coat protein and transcription activator protein gene homologue.

The inferred amino acid sequences of EGV1 and EGV2 Rep proteins contained canonical rolling circle replication (RCR) motifs which, in addition to being present in all known geminivirus Reps, are highly conserved among many other rolling circle replicons (Ilyina and Koonin 1992) (Supplementary Fig. S2). Also, the inferred EGV1 and EGV2 Rep homologues contain an apparent dNTP-binding site, which, in free-living geminiviruses, is potentially associated with helicase activity (Supplementary Fig. S2) (Choudhury et al. 2006). The EGV1 and EGV2 Rep proteins also do not harbour a canonical retinoblastoma binding motif LxCxE (Arguello-Astorga and Ruiz-Medrano 2001) but contain a GRS-like domain that has been identified in geminivirus Reps and putatively contributes to the structural integrity of the Rep protein (Nash et al. 2011) and a possible ‘helix 4’ retinoblastoma interaction motif (ALxIIRExxP between positions 147 and 156) that has been previously identified in begomovirus Reps (Arguello-Astorga et al. 2004).

### 3.3 EGV1 and EGV2 sequences are likely integrated within the *D. alata* genome

EGV1 and EGV2 were detected among asymptomatic *D. alata* plants grown from true seeds under insect-proof conditions (Supplementary Table S2). Moreover, EGV1 and EGV2 were detected in all asymptomatic *D.* *alata* plants that were collected all over the world (Supplementary Table S2). These EGV sequences were probably neither infectious nor insect transmissible because they lacked any evidence of capsid, movement or transcription activator protein encoding genes. Although capsid protein expression is absolutely required for insect transmission (Hanley-Bowdoin et al. 2013), these other genes are collectively required by free-living begomoviruses for infectivity.

Although random primed RCA is a standard technique used in the isolation and cloning of geminivirus genomic DNA from geminivirus infected plants (Haible, Kober, and Jeske 2006), EGV1 or EGV2-like amplicons could not be obtained from *D. alata* plants even using a cocktail of random and specific primers that had previously been successfully used for detecting EGV1 or EGV2 by PCR. This suggested that no circular episomal EGV1 or EGV2-like molecules were present within DNA extracts of *D. alata* plants (Supplementary Fig. S3).

Given (1) that the EGV1 and EGV2 sequences are apparently stably inherited, (ii) that they are not amplifiable by RCA, (iii) that very similar sequences are present in a range of asymptomatic *D. alata* plants sampled worldwide, and (iv) that they have no discernable coat, movement, or transcription activator protein genes (which suggests they are not likely to be insect transmissible or even infectious), we hypothesized that EGV1 and EGV2 were integrated within the *D. alata* genome. We furthermore postulated that one or several geminiviral progenitors of these sequences were likely to have become integrated into the genome of an ancestor of the Asian yam species belonging to the Enantiophyllum clade of the *Dioscorea*.

**Figure 1.**
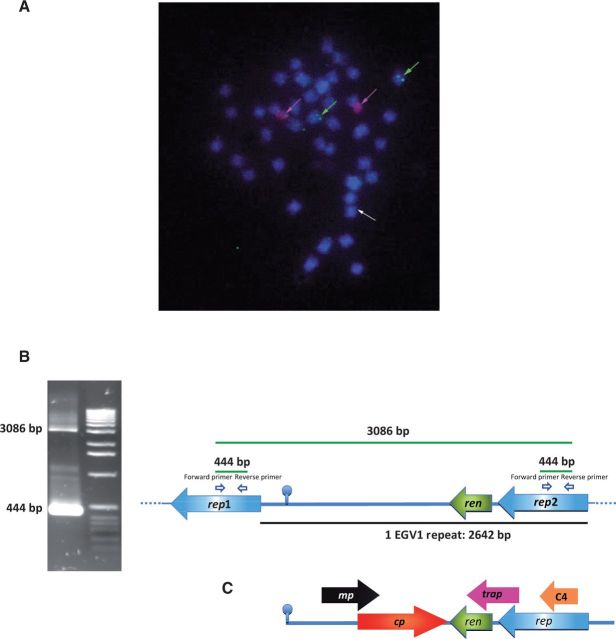
(A) FISH on *D. alata* chromosomes with a 2.6 kb EGV1 probe from *D. alata* (detected in green) and a rDNA 45 S probe used as control (detected in red). Chromosomes are counterstained with DAPI, in blue. Scale bar = 5 µm. The green arrows indicate four hybridization signals potentially corresponding to the presence of tandem repeats of EGV located on both chromatids of two chromosome pairs. The white arrows indicate faint signals potentially corresponding to the presence of copies of the *rep* gene scattered around the genome of *D. alata*. (B) Long template PCRs enabled the amplification of two DNA fragments (444 bp and 3,086 bp) from *D. alata* acc. 313. Sequencing of 444 and 3,086 fragments revealed a partial tandem repeat genomic organization with two partial *rep* genes, a *ren* gene, and an intergenic region containing a GC-rich stem carrying a nonanucleotide loop TAATATTAC. The size of one repeat is 2,642 bp. (C) Representation of a typical linearized begomovirus genome in the virion sense orientation starting from the origin of replication (mp, movement protein; cp, capsid protein; ren, replication enhancer; trap, transactivator protein; rep, replication-associated protein; C4 ORF has been shown to suppress transcriptional gene silencing).

We tested the first hypothesis by checking for integration of the EGV1 sequences by FISH. Fluorescent green hybridization signals were detected unequivocally on two out of the forty *D. alata* chromosomes in all metaphase cells examined (Fig. 1A). Clear pairs of dots on two different pairs of chromosomes were detected at similar intensities in multiple cells using multiple root-tip preparations (green arrows in Fig. 1A). Several other chromosomes also showed very faint (but clearly visible) hybridization signals (white arrows in Fig. 1A), which possibly corresponded to (1) non-specific probe hybridization, (2) small dispersed EGV1 sequence fragments, or (3) more distantly related geminivirus-like sequence integrons such as EGV2. The brightness of the FISH signals seemed to be similar to the brightness previously obtained with known endogenous geminivirus (Kenton et al. 1995; Ashby et al. 1997) and endogenous Banana streak virus sequences (Harper et al. 1999), which were also both found to be inserted as multiple copies. Thus, it is supposed that the EGV1 elements may be organized as complex repetitive inserts of EGV1 that are greater than 2.6 kb in length.

### 3.4 The EGV1 sequences likely occur as long tandem repeats

To test the hypothesis of multiple EGV1 repeats existing in the *D. alata* acc. 313 genome, we set up ‘*rep-rep’* and ‘*ren-ren’* long template PCRs to amplify multiple copies of this sequence. Fragments of approximately 0.4 kb and 3.1 kb (‘*rep-rep’*; Fig. 1B) and 0.6 kb and 3.2 kb (‘*ren-ren’*) were obtained, confirming the likely presence of tandem repeats of 2.6 kb EGV1 integrons within the *D. alata* acc. 313 genome (Fig. 1B).

A second line of evidence that there were multiple copies of EGV1 inserted in the *D. alata* genome came from an analysis of EGV1 sequence diversity. Alignment and pairwise analysis of nucleotide polymorphisms within twenty-four copies of the 402 bp *rep* EGV1 fragments obtained from the ‘*rep-rep’* and ‘*ren-ren’* long PCRs indicated that there exist at least eighteen genetically distinct copies of the EGV1 *rep* gene integrated within the *D. alata* genome (Supplementary Fig. S4). This result suggests that the copies form part of tandem repeat arrays of EGV1 sequence that may exceed 23 kb (18 × 2.6 kb/2 loci).

### 3.5 The *rep* genes of EGV1 and EGV2 have likely been functionally transcribed post-integration

**Figure 2.**
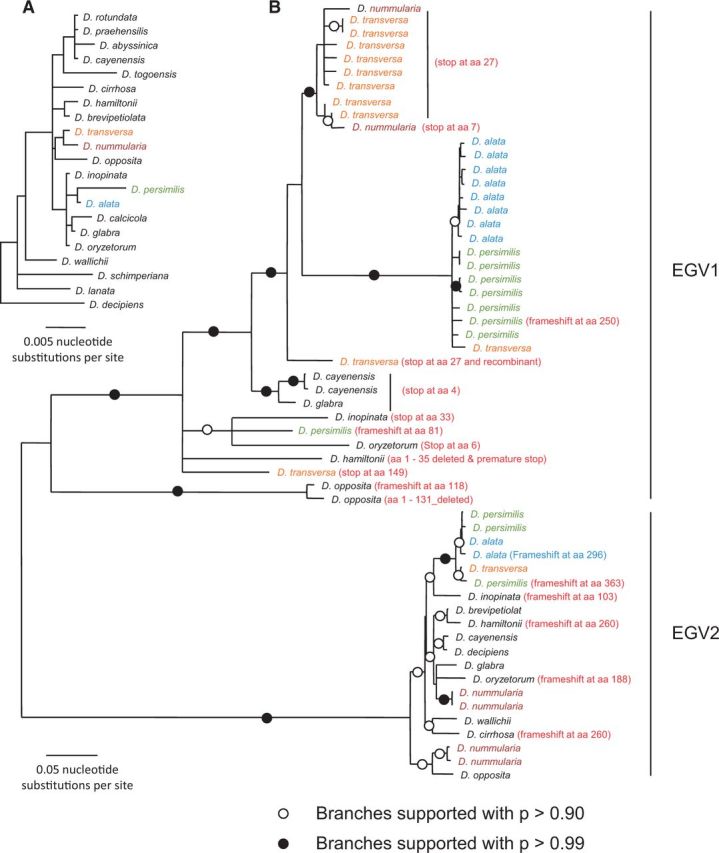
Maximum likelihood trees of (A) yam *matK* & *rbcL* concatenated sequences from Enantiophyllum *Dioscorea* spp. where EGV1 and EGV2 were detected (branches with less than 50% bootstrap support have been collapsed) and (B) thirty-seven EGV1 and twenty EGV2 replication-associated protein gene (rep) nucleotide sequences derived from fourteen different yam species. Indicated in red are the positions of mutations that might impact Rep gene expression from some of the EGV sequences.

Of the fifty-seven EGV *rep* gene copies derived from the various *Dioscorea* species, twenty-nine (fifteen from EGV1 sequences and fourteen from EGV2 sequences; Fig. 2) contained neither frameshift mutations nor premature stop codons. Canonical RCR motifs were present in twenty-one of twenty-nine of these genes, which suggests that they could possibly still be capable of encoding functional proteins. In addition, the fact that *rep* containing transcripts of EGV1 and EGV2 were identified in our screening of *D. alata* and *D.* *rotundata* EST datasets indicates that the *rep* genes of at least some of the EGV1 and EGV2 integrons are transcribed in at least some of the *Dioscorea* species.

To further test the hypothesis that EGV sequences are being functionally transcribed, we analysed siRNA sequences from *D. alata*. Virus-derived siRNAs naturally accumulate in virus-infected plants as a consequence of plant RNA silencing-based antiviral defences (Voinnet 2005). A defining characteristic of an active silencing response is the dicer (or dicer-like) enzyme-mediated degradation of double-stranded virus-derived RNA into 21–24-nt-long RNA fragments (Pooggin 2013; Pumplin and Voinnet 2013). The occurrence of EGV-associated 21, 22, and 24 nt siRNAs implies that at least some of the EGV sequences that are present in the *D. alata* genome are transcribed and processed by distinct Dicer-like (DCL) enzymes both within the nucleus (the 24 nt size class) and the cytoplasm (the 21 and 22 nt size classes) to produce the different siRNA size classes (Pooggin 2013; Pumplin and Voinnet 2013).

**Figure 3.**
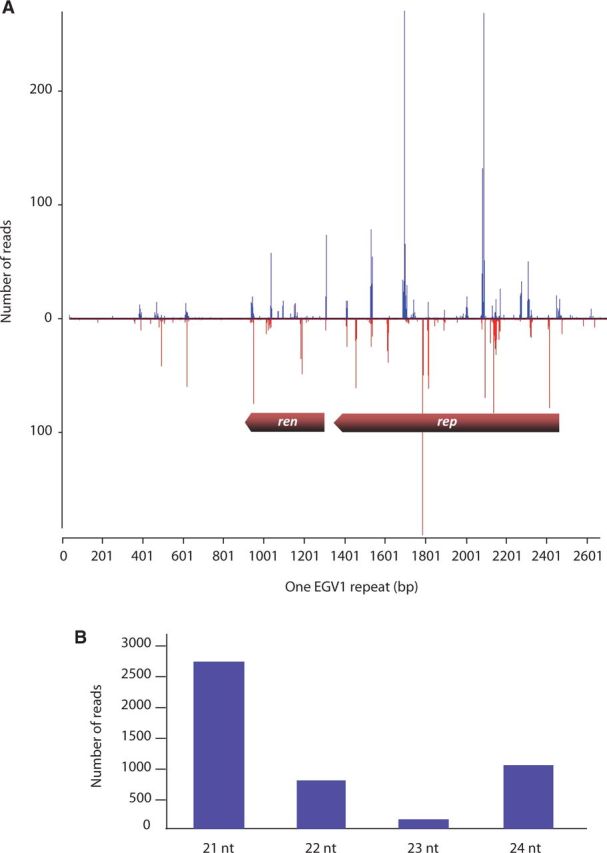
(A) EGV1 sequence coverage following Illumina-based siRNA analysis of the *D. alata* acc. W10-223 plant. The graphs plot the number of 21–24 nt siRNA reads at each nucleotide position of *ren* and *rep*; bars above the axis represent sense reads starting at respective positions; those below the antisense reads ending at respective positions. The genomic organization of EGV1 (*rep* and *ren* genes) is schematically shown. (B) Size distribution of siRNAs mapping to EGV1 sequence.

A total of 15,365,074 raw Illumina sequence reads were generated from *D. alata* acc. W10-223. Seven of the >100-bp long contigs that were obtained by *de novo* assembly showed significant degrees of similarity to begomoviruses based on BlastX searches (58.9–77.5% identity). All seven of these contigs corresponded with begomovirus *ren* and *rep* genes. Although only fifteen of the Illumina reads mapped to the 2.9 kb EGV2 sequence, a total of 4,757 reads mapped to the 2,642 bp EGV1 sequence (Fig. 3). Although the discovery of siRNAs corresponding to both EGV1 and EGV2 sequences implies that both classes of integrated sequences are transcriptionally active, the much greater depth of siRNA coverage observed for EGV1 relative to EGV2 strongly suggests that EGV1 is more transcriptionally active than EGV2. In addition, this result potentially indicates that the tandem EGV1 integron repeat sequences might play a key role in the maintenance of the silencing response.

In total, 65.5 per cent of the EGV1 sequence was covered at an average depth of 38X (Fig. 3). The distribution of reads across EGV1 was highly heterogeneous, with most (92%) corresponding to portions of *ren* (coverage 86.7%, average depth of coverage of 40X) and *rep* (coverage 75.8%, average depth of coverage of 74X) genes. Apart from a 250-bp region (positions 401–751; 5.5% of the reads) nearby the *ren* gene (Fig. 3) that does not apparently encode any protein, very few of the siRNA reads mapped to the non-coding portions of EGV1 (2.5%). The high density of siRNA reads corresponding to the *rep* and *ren* genes of EGV1 and low density of siRNA corresponding to the remainder of the EGV sequences are consistent with the hypothesis that the *rep* and *ren* genes are being specifically, and potentially functionally, transcribed.

The distribution of size classes of the siRNAs corresponding to EGV1 sequences was clearly enriched for 21, 22, and 24 nt siRNAs (Fig. 3). This siRNA size-class distribution is indicative of the EGV1 sequences being targeted by both post-transcriptional gene silencing (as indicated by the 21 nt and 22 nt size classes that are, respectively, produced by the antiviral dicers, DCL4 and DCL2) and transcriptional gene silencing (as indicated by the 24 nt size class that is presumably produced by the DCL3 dicer (Aregger et al. 2012)). The fact that silencing of both types are specifically targeting the *rep* and *ren* genes of EGV1 provides further evidence that these genes are transcribed.

### 3.6 Evidence that the Rep proteins of EGV1 and EGV2 have been functionally translated post-integration

To test whether either EGV1 or EGV2 Rep amino acid sequences might have been functionally translated within yam genomes, we attempted to determine whether, following their integration, the *rep* gene sequences displayed any evidence of having accumulated fewer non-synonymous substitutions (i.e. nucleotide substitutions within codons that alter encoded amino acid sequences) than would be expected under neutral evolution. Decreased rates of non-synonymous substitution relative to synonymous substitution are strongly indicative of natural selection favouring the maintenance of functional amino acid sequences.

Using a random-effects phylogenetic model of selection, we detected very strong evidence of purifying selection along the branch separating EGV1 and EGV2 (Table 2). As has been noted for the two distinct GRD elements found integrated into the genomes of some *Nicotiana* species, this signal of purifying selection is consistent with two distinct EGV integration events (Murad et al. 2004). If the branch of the phylogenetic tree separating the EGV1 and EGV2 sequences represents ‘free living’ ancient begomovirus-like viruses, then negative selection detected among the nucleotide substitutions that map to this branch likely reflect the action of negative selection such as that detectable within contemporary begomovirus *rep* sequences (Lima et al. 2013). If, however, the geminivirus sequence that originally became integrated within an ancestral yam genome was a common ancestor of EGV1 and EGV2, then the negative selection detected on the branch of the phylogenetic tree separating the EGV1 and EGV2 sequences could also reflect post-integration selection favouring the maintenance of functional Rep proteins.

**Table 2.** Selection analyses results.

EGV1	EGV2	Connecting branch	*P* value for neg. selection	*ω*_1_	*P*_1_
BG1	BG2	FG	0	0.027	0.76
BG1	FG	BG2	0.021	0	0.32
FG	BG1	BG2	0.00029	0.18	0.80
FG	FG	BG1	0.00013	0	0.40

For each test, each clade was assigned to a foreground partition (FG) or one of two background partitions (BG1 and BG2). We report *P* values for significance tests for purifying selection along foreground lineages and the parameter point estimates for the foreground partition (w1 = dN/dS of negateively evolving codon sites and P1 = the proportion of all codon sites that are negatively evolving) under the alternative model (which allows purifying selection).

This latter possibility is particularly plausible in that, unlike with the *Nicotiana* geminivirus-like integrons, our selection analysis also detected both strong evidence of purifying selection post-dating the EGV1 most recent common ancestor (MRCA, *P* = 0.00029), and moderate evidence for purifying selection post-dating the EGV2 MRCA (*P* = 0.021; Table 2). Crucially, our approach did not find similar evidence of post-integration negative selection within the *Nicotiana* geminivirus-like integrons (minimum *P* = 0.44; Supplementary Table S4) indicating that a previous failure to detect evidence of selection in these sequences was not simply due to the application of a less-powerful analytical approach (Murad et al. 2004).

Regardless of whether EGV1 and EGV2 share a common integrated ancestor, evidence of natural selection actively favouring the maintenance of both EGV1 and EGV2 derived Rep amino acid sequences indicates that functional Rep proteins were probably translated for prolonged periods following the integration of these sequences.

**Figure 4.**
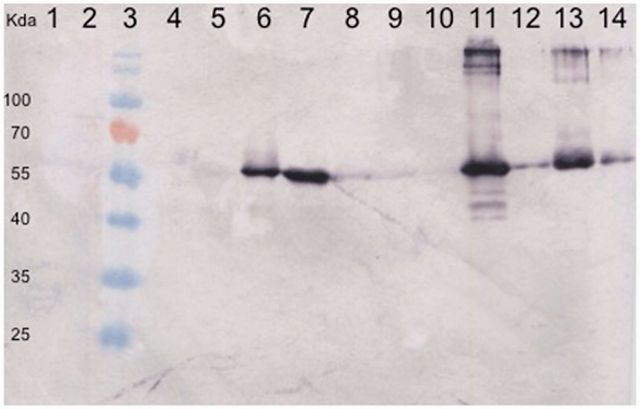
Western blot on the total protein extracts of several *Dioscorea* species, using an antibody directed to a Rep peptide of EGV1. Lanes: (1) proteins extracted from a turnip plant infected by Cauliflower mosaic virus (CaMV); (2) a Tomato plant infected by TYLCV; (3) ladder; (4) *D. trifida* (accession no. 64); (5) *D. sansibarensis* (accession no. 269); (6) *D. bulbifera* (accession no. 272); (7) *D. dumetorum* (accession no. 47); (8) *D. togoensis* (accession no. 114, seedling); (9) *D. praehensilis* (accession no. 255); (10) *D. rotundata* (accession no. 118, seedling); (11) *D. nummularia* (accession no. 335); (12) *D. alata* (accession no. 297, seedling); (13) *D. alata* (accession no. 313 seedling); (14) *D. alata* (accession no. 402 seedling). No cross-reactivity was detected in the turnip/CaMV, tomato/TYLCV, *D. trifida,* and *D. sansibarensis* samples.

The tantalizing possibility that the Rep protein may still be translated in some contemporary *Dioscorea* species was therefore tested by Western blotting. Anti-Rep antibodies were produced against a synthesized oligopeptide selected from the predicted Rep of EGV1. It is noteworthy that this oligopeptide did not display any significant similarity to either (1) any translated yam sequences that are currently available within public sequence databases (2,781 nucleotide sequences in GenBank and 48,527 in the EST database) or (2) any eukaryotic or prokaryotic proteins in any of the publically available NCBI sequence databases: in both cases BlastX or tBlastN *E*-values that were obtained were uniformly >10^−1^. An approximately 55-kDa protein was detected in *D.* *bulbifera, D.* *dumetorum, D.* *nummularia*, and the three *D. alata* seedling accessions. EGV1 Rep expression was also evident (albeit to a lesser degree) in *D.* *togoensis, D.* *praehensilis,* and *D.* *rotundata* (Fig. 4). In addition, this protein was not detected either in *D.* *trifida* and *D.* *sansibarensis* or in the two negative controls, including the tomato plant infected with TYLCV-mld (which was likely to contain both tomato and TYLCV-mld proteins; Fig. 4 and Supplementary Fig. S5). Although the theoretical mass of the Rep protein deduced from the EGV1 Rep sequence is expected to be 43 kDa, such differences between estimated and actual protein masses are frequently observed and are usually attributable to post-translational modifications (Shirai et al. 2008). Another possibility is that we have detected a protein that is unrelated to the EGV1 Rep: a protein that while present to varying degrees in all of the EGV1 containing species that we tested is apparently absent in some (but not all) of the yam species where EGV1 sequences remained undetected.

Although not entirely definitive, these experiments nevertheless provide additional evidence that the integrated EGV1 rep genes are both still being expressed in multiple different *Dioscorea* species and evolving under a degree of purifying selection. This suggests that at least some of the Rep proteins that are encoded by EGV1 *rep* genes are likely still functionally active.

### 3.7 Phylogenetic relationships between EGV1, EGV2, and the geminiviruses

**Figure 5.**
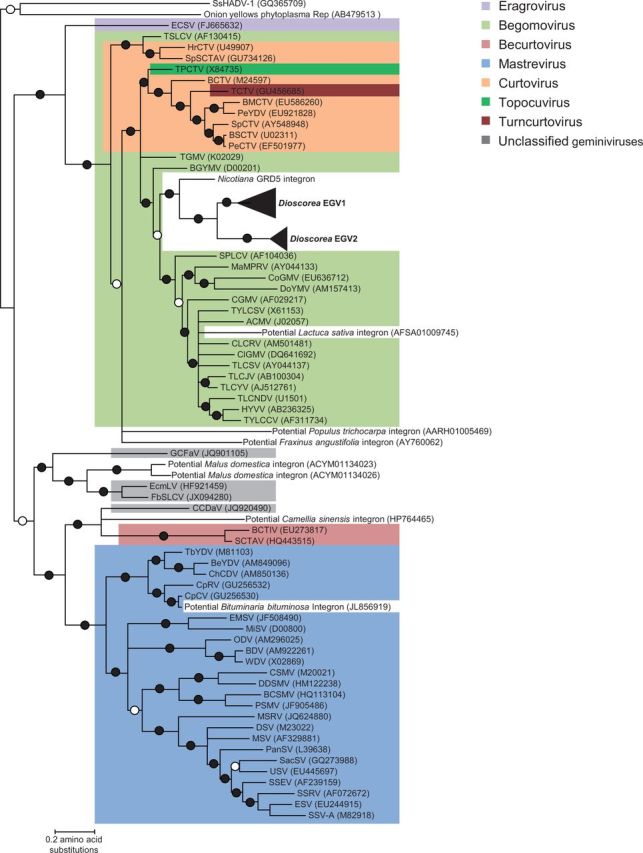
Maximum likelihood trees of Rep amino acid sequences encoded by the *rep* gene of representatives of EGV1 and EGV2 and sixty-nine other viruses representing the broadest breadth of currently sampled geminivirus diversity, including endogenous geminivirus sequences clearly apparent within the recently published genome sequences of *L. sativa* (common lettuce, GenBank accession: PRJNA68025), *M. domestica* (common apple tree, GenBank accession: PRJNA28845) *P. trichocarpa* (black cottonwood tree, GenBank accession: PRJNA17973), *Fraxinus angustifolia* (narrow-leafed ash, GenBank accession: AY760062), *Camellia sinensis* (tea plant, GenBank accession: HP764465), and *Bituminaria bituminosa* (Arabian pea, GenBank accession: JL856919). Branches with a filled dot have >99 per cent posterior probability support, whereas those with an empty dot have >95 per cent posterior probability support. All branches with less than 80 per cent posterior probability support have been collapsed.

As *rep* and *ren* were the only EGV genes with obvious homology to geminiviruses genes, we focused on these to explore the possible evolutionary relationships between the EGVs and geminiviruses (Fig. 5 for Rep and Supplementary Fig. S6 for Ren). The EGV1 and EGV2 Rep (predicted proteins of 372 and 371 aa in length, respectively) and Ren sequences from *Dioscorea* species both clearly form sister clades nested within larger begomovirus clades, which strongly suggests that the EGV1 and EGV2 sequences were derived from ancient begomoviruses.

### 3.8 Distribution of EGV1- and EGV2 sequences among members of the *Dioscoreacea* family

**Figure 6.**
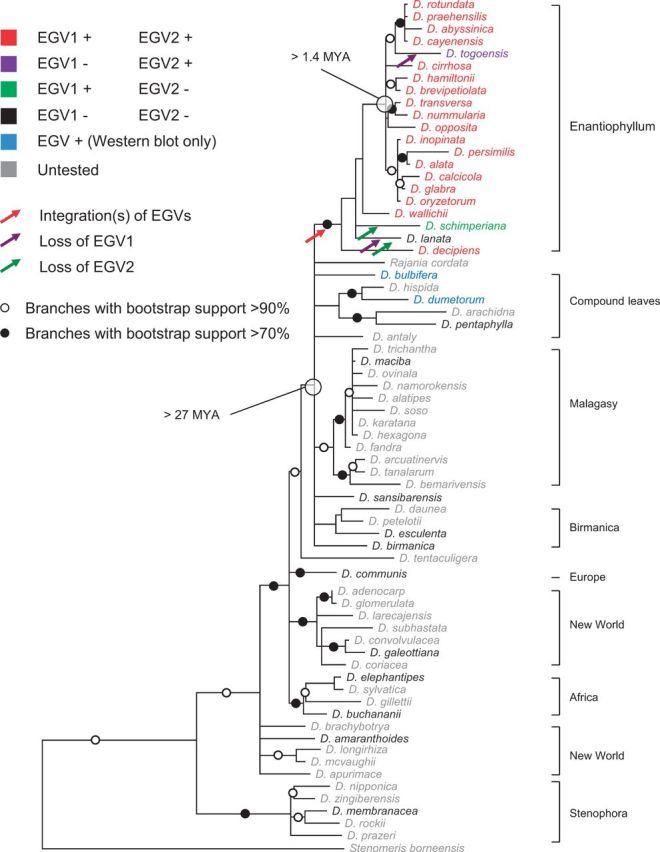
Maximum likelihood tree describing the evolutionary relationships between African and Asian yam species from the Enantiophyllum clade and other representative species from other clades of *Dioscoreacea* based on *rbcL* and *matK* nucleotidic nucleotide sequences. Branches associated with a filled dot have bootstrap support above 90 per cent whereas those with an unfilled dot have bootstrap support above 70 per cent. All branches with less than 50 per cent bootstrap support have been collapsed.

Using PCR, EGV1 and EGV2 *rep* sequences were only detected in yam species belonging to the Enantiophyllum clade of *Dioscorea*, all of which originate from Asia and Africa (Table 1). Although both EGV1 and EGV2 sequences were detectable within nineteen yam species, EGV1 sequences were additionally detectable in *D.* *schimperiana* and EGV2 sequences were additionally detectable in *D.* *togoensis* (Table 1). Based on these PCR results, *D. lanata* apparently lacks both EGV1 and EGV2, suggesting that this yam lineage either lost the integrated EGV sequences or contains EGV sequences that are simply not detectable with the PCR primer sets that we used to screen for these EGVs. This latter possibility is supported by the fact that *D.* *bulbifera* and *D.* *dumetorum* of the compound leafed (CL) clade of *Dioscorea* also tested negative for the EGVs by PCR but positive for the presence of EGV1-derived Rep protein expression using Western blotting (Fig. 4). This result strongly suggests that the integration of these EGV sequences predated the divergence of the Enantiophyllum and CL clades of the *Dioscorea* (Fig. 6).

The CL clade includes species with compound leaves and the species *D.* *antaly* and *D.* *bulbifera* which have simple leaves (Fig. 6). Interestingly, a fossilized compound *Dioscorea* leaf dated to approximately 27 MYA (Pan, Jacobs, and Currano 2014) implies that the *Dioscorea* lineage with compound leaves diverged from the Enantiophyllum clade prior to this date and, therefore, that the MRCA of the Enantiophyllum clade of *Dioscorea* may have existed prior to 27 MYA.

A second less parsimonious scenario would be based on two independent EGV integrations: one in the Enantiophyllum clade and the other in the CL clade. The presence of EGV1 and EGV2 sequences in almost all tested African and Asian Enantiophyllum section species (including the pre-ennobled *D. praehensilis* and wild *D.* *minutiflora*) suggests that the geminiviral integration event(s) predated the diversification of Asian and African Enantiophyllum lineages, which may have occurred during the late Pliocene to early Pleistocene, 4.3–1.4 MYA when dry woodlands replaced rainforests that previously extended to a latitude of 20° N (Feakins et al. 2013). In this scenario, the MRCA of the Enantiophyllum section *Dioscorea* species could credibly have existed between 1.4 and 27 MYA.

### 3.9 Duplication and diversification of the yam EGVs following their integration

To determine whether the EGV sequences have co-diverged with the *Dioscorea* species of the Enantiophyllum clade, we compared the phylogenies of EGV1 and EGV2 *rep* sequences with that of the *Dioscorea* species from which they were isolated (as determined using concatenated *rbc*L and *mat*K sequences). If EGV1 and EGV2 sequence duplications (two, two, eight, eight, and eleven EGV1 copies in *D. opposita* acc. 265, *D. cayenensis* acc. 1, *D. alata* acc. 313, *D. persimilis* acc. 271, and *D. transversa* acc. 336, respectively; and two, three, and four EGV2 copies in *D. alata* acc. 313, *D. persimilis* acc. 271, and *D. nummularia* acc. 206, respectively) had only occurred at the time when these sequences became integrated (i.e. prior to the divergence of the various EGV containing species), then one would expect that genetically distinct endogenous sequences sampled from a particular *Dioscorea* species would almost always be less closely related to one another than to endogenous sequences sampled from other *Dioscorea* species (Supplementary Fig. S7A). If, however, endogenous sequence duplication events were interspersed with *Dioscorea* speciation events, then one would expect that genetically distinct endogenous sequences isolated from a particular *Dioscorea* species would frequently be more closely related to one another than to endogenous sequences sampled from other *Dioscorea* species (Supplementary Fig. S7B).

It is evident that both the EGV1 and EGV2 sub-trees better match this latter expectation (Fig. 2), indicating that EGV1 and EGV2 duplication events have likely occurred intermittently with *Dioscorea* speciation events. Further, the occurrence of very closely related groups of both EGV1 sequences in *D. opposita*, *D. cayenensis*, *D. persimilis*, *D. transversa,* and *D. alata*, and EGV2 sequences in *D. nummularia*, *D. persimilis* and *D. alata* suggests that both the EGV1 and EGV2 duplication events have likely occurred up until very recently in the evolutionary histories of these various *Dioscorea* species.

It should be noted, however, that hybridization between the different Enantiophyllum species may also have blurred the two scenarios described above. For instance, genetically distinct endogenous sequences sampled from *D. transversa* are either less or more closely related to one another than they are to endogenous sequences sampled from *D. nummularia*, *D. alata,* and *D. persimilis*. These relationships can be explained by the fact that *D. transversa* shares a common genetic background, probably through hybridization, with the southeast Asian-Oceanian species *D. nummularia*, *D. alata* and *D. persimilis* (Malapa et al. 2005). In addition, EGV sequences sampled from *D. alata* and *D. persimilis* are very closely related to one another: a fact supporting the hypothesis that *D. persimilis* is a progenitor of *D. alata* (Mignouna et al. 2002).

## 4 Conclusion

Even though the integration of DNA viruses into host genomes has been repeatedly reported in bacterial and animal systems (Feschotte and Gilbert 2012), there are still few reports of horizontal transmission of ssDNA virus sequences into the nuclear genomes of plants (Feschotte and Gilbert 2012). For instance, the integration of GRD into the nuclear genome of an ancestral *Nicotiana* remains the only ssDNA virus integration event into a plant genome that has been definitively proven so far (Bejarano et al. 1996; Ashby et al. 1997). In addition, traces of geminiviral sequences have also been reported from several recently sequenced plant genomes (*L. sativa*, *M. domestica, C. canephora,* and *P. trichocarpa*) (Liu et al. 2011; Martin et al. 2011). Unfortunately, the integration status of these geminivirus-like sequences has so far not been experimentally confirmed, and one cannot discount the possibility that these examples simply represent episomal viral contamination. The experiments we have described here, however, provide proof of a second group of endogenous geminivirus-like sequences present in various yam species and suggest that endogenous geminiviruses may be more common in plant genomes than has previously been appreciated. The growing availability of large numbers of plant genome sequences combined with enhanced computational tools geared to detect integrated viral genomes will likely expedite the discovery of many other such EGVs in the coming years.

There are a number of possible reasons why the endogenization and expression of a geminivirus *rep* gene might have been (and might still be) selectively advantageous to a host plant species. Such genes might confer virus resistance such as that demonstrated by transgenic plants that express the oligomerization domains of Rep proteins (Chatterji, Beachy, and Fauquet 2001). In fact, just the transcription of geminivirus-related sequences within transgenic plants can also provide geminivirus resistance via the induction of innate host silencing-based antiviral defence mechanisms (reviewed in Shepherd, Martin, and Thomson (2009)). Endogenized *rep* sequences could therefore simply represent a natural form of genetically engineered virus resistance. The protection provided by such endogenized geminivirus sequences would, however, potentially only be effective against viruses with *rep* genes that are genetically very similar to those of the endogenized *rep* genes (Antignus et al. 2004).

Another possibility is that the endogenous *rep* sequences may have been recruited by the host to perform a particular cellular function. Geminivirus Rep proteins are multifunctional and could provide cellular functions including the induction of DNA replication (Nagar, Hanley-Bowdoin, and Robertson 2002), cell cycle arrest, and transcription (Hanley-Bowdoin et al. 2013). It is not entirely implausible that, following integration, one of these functions provided the selection pressure needed to maintain functional EGV Rep expression.

Finally, it is possible that the integrated geminivirus sequences are simply parasitizing the various yam species in which they are found. Signals of selection detectable within these genes likely reflect evolutionary pressures favouring the persistence of EGV1 and EGV2 sequences within yam genomes. As with the GRD3 family of geminivirus-like sequences integrated within some *Nicotiana* genomes, the EGV sequences described here have some structural features in common with Helitrons: a group of autonomous, potentially parasitic, transposable elements found within the genomes of plants and other eukaryotes that replicate via a rolling circle mechanism (Kapitonov and Jurka 2001). These structural features include a twenty nucleotide palindromic hairpin loop sequence (found in both the *Nicotiana* GRD and *Dioscorea* EGV sequences) with a downstream CTRR motif (found in the *Nicotiana* GRD sequences) and the expression of a Rep protein with both DNA helicase and rolling-circle replication initiation activities (found in the *Dioscorea* EGV sequences) (Murad et al. 2004).

Given the growing number of instances where *rep* genes derived from small ssDNA viruses (including unclassified ssDNA viruses (Belyi, Levine, and Skalka 2010; Kapoor, Simmonds, and Lipkin 2010), circoviruses (Belyi, Levine, and Skalka 2010), geminiviruses (Bejarano et al. 1996), and parvoviruses (Belyi, Levine, and Skalka 2010; Kapoor, Simmonds, and Lipkin 2010)) have been found integrated into various eukaryote genomes, it is possible that there is some general feature of rolling circle replicons that enables their *rep* genes to both invade and persist within host genomes. In the case of each of the two yam integrons it is likely that, after the initial integration of a full or partial begomovirus genome into a chromosome within the nucleus of a totipotent plant cell, a process of endogenization occurred. This might have initially involved the pseudogenization or loss of all the integrated virus’ genes other than *rep* and *ren*, followed by the *rep*-mediated duplication and expansion of the integron sequences and, finally, the re-orientation of Rep towards a transposition function. Access to a variety of complete yam genome sequences will be critical in efforts both to determine the complete spectrum and distribution of EGV sequences within yam genomes and to identify those EGV copies that are most likely to still have some degree of biological functionality. Experimental characterization of these particular EGV copies would then indicate whether the EGVs found in yam are indeed Helitron-like elements.

## Data available at NCBI GenBank accession numbers

EGV1_*rep* genes: KJ629184–KJ629216, EGV2_*rep* genes: KJ629217–KJ629236, m*at*K partial genes: KJ629237–KJ629250, r*bc*L partial gene: KJ629251–KJ629264, ‘r*en-ren’* long template PCRs fragments: KJ629265–KJ629274, ‘*rep-rep’* long template PCRs fragments: KJ629275–KJ629281, and EGV1 and EGV2 units: KJ629282–KJ629285

## Supplementary Material

Supplementary Table S1
