# Supplementary material for: The genomes of many yam species contain transcriptionally active endogenous geminiviral sequences that may be functionally expressed
Source: Virus Evol. 2015 May 26;1(1):vev002. doi: 10.1093/ve/vev002 (PMC5014472; doi:10.1093/ve/vev002)
Supplement: Supplementary Table S1 [file ve_vev002_index.html]

Supplementary Data | Virus Evolution

## Supplementary Data

files

- Supplementary Data - pdf file
