# Supplementary material for: The genomes of many yam species contain transcriptionally active endogenous geminiviral sequences that may be functionally expressed
Source: Virus Evol. 2015 May 26;1(1):vev002. doi: 10.1093/ve/vev002 (PMC5014472; doi:10.1093/ve/vev002)
Supplement: Supplementary Table S1 [file Supplementary_Data.pdf]

Supplementary Figure 1. (A) Annotated 2.6 Kb endogenous geminivirus nucleotidic sequence of D. alata. (B). Annotated 2.1 Kb endogenous geminivirus nucleotidic sequence of D. alata.

Supplementary Figure 2. Alignment of the putative Rep sequences of EGV1 and EGV2 with the Rep of six representative geminiviruses (Tomato golden mosaic virus (TGMV), Maize streak virus (MSV), Eragrostis curvula streak virus (ECSV), Bean golden mosaic virus (BGMV), Beet curly top virus (BCTV) and Tomato pseudo-curly top virus (TPCTV)). RCR motifs (I, II, III and GRS) and SF3 helicase motifs (Walker A, B and C), which are conserved in geminiviruses, are highlighted in red.

Supplementary Figure 3. Rolling circle amplification. Lanes: 1) Ladder; 2) pUC19; 3) African cassava mosaic virus (ACMV); 4) Dioscorea badnavirus; 5) Water; 6) Dioscorea alata (acc. #313, seedlings); 7) Dioscorea tranversa (acc. #336).

Supplementary Figure 4. Maximum likelihood tree of eighteen 402 bp partial rep sequences obtained using the “ren-ren” and “rep-rep” primer pairs.

Supplementary Figure 5. (A) Western blot on the total protein extracts of several Dioscorea species, using an antibody directed to a Rep peptide of EGV1 and (B) nitrocellulose membrane stained with Ponceau S dye for protein detection during western blotting. Lanes: 1) Proteins extracted from a turnip plant infected by Cauliflower mosaic virus (CaMV); 2) a Tomato plant infected by Tomato yellow leaf curl virus (TYLCV); 3) Ladder; 4) Dioscorea trifida (accession#64); 5) Dioscorea sansibarensis (accession#269); 6) Dioscorea bulbifera (accession#272); 7) Dioscorea dumetorum (accession#47); 8) Dioscorea togoensis (accession#114, seedling); 9) Dioscorea praehensilis (accession#255); 10) Dioscorea rotundata (accession#118, seedling); 11) Dioscorea nummularia (accession#335); 12) Dioscorea alata (accession#297, seedling); 13) Dioscorea alata (accession#313 seedling); 14) Dioscorea alata (accession#402 seedling). No cross-reactivity was detected in the turnip/CaMV, tomato/TYLCV, D. trifida and D. sansibarensis samples.

Supplementary Figure 6. Maximum likelihood trees of replication enhancer protein (Ren) amino acid sequences (JTT substitution model) of EGV1 and representative geminiviruses, from the genera begomovirus topocuvirus and curtovirus. Numbers associated with branches indicate degrees of bootstrap support (100 replicates) for those branches.

Supplementary Figure 7. Duplication and diversification of the yam EGVs following their integration. Two scenarios resulting in contrasting patterns of endogenous sequence diversity are expected and presented. (A) The endogenous sequences were only duplicated (indicated by red arrows) either at or very close to the time when the integration event occurred (indicated by green arrow), or (B) The endogenous sequences are duplicated for prolonged periods post-integration such that duplication and speciation events (indicated by blue arrows) are interspersed. In (A) endogenous sequences sampled from one species will usually be more closely related to endogenous sequences from different species than they are to other genetically distinct endogenous sequences sampled from the

same species. In (B) genetically distinct endogenous sequences sampled from the same species should frequently be more closely related to one another than they are to endogenous sequences sampled from different species.

A- EGV1 annotated sequence from *D. alata* acc. 313

Virion strand origin of replication | Inverted repeat sequence

TAATATTACACGGATGGCCGCCCCGCACTGTACTGCCACGTGGCGCATTTTGATAGGTCCACGCTAAAGATGCTGTACCC 80

GATTGTCTCCCACGGTAAAGATGCTGTACCCGATTGTCTCCCACCGTACATCCACGCGCCATCATGGCATTGGTCCATTC 160

AAATACAAACCGGCGCCCGAAATTTAATATGCCGAGAAAAATAAATAGGCGCCCGAATTGATGTGTTCCTTTCTCTGGCG 240

TGGGTCCCACTATATTCAAAAATAATAATAAACAAACCATTTAATAGTGTTGTTGGTACAAGAGTGCGCCAAATTGTTGA 320

AATATATTCAAACACTCTACAGCGCCGTATGATGACTACATCAATATCGAGAAACTTGGACCGGAAGGCTAATTATTATA 400

TAAGGCGATGAACCAGAGTCTCCTCTCAGGCAAGATGGATAAGGAAGAAATAATGGCCGTATACCTGCTCTTCCTCAGAT 480

ACGGACCATCAGAAATAATTAAATGTTTGCACGGCATTTGCATCGCCGAAGCAATTGAGGAAAATCCACTACCTCTTCTG 560

GAATATGTCAACTCACTTCAGAAGCATCAAGAAGCCGTGTACAGAATGCTCCGCGAAGCCGGGAAAACTAGTAGTGAAGT 640

AGAAGAAGTCACTGAGGCTTATACCGTCGTTGACAGATCCGGCAAGAAGAAAAAAATGAAAGTGGAAGACCTCGGCGAAG 720

TTGGTACCTCGAAGGGAGTTGTTTCTACCGGATGGTGACTGTCATACATCCGGCGTCGTATATTTATATTAGTATTAGTA 800

TTGTAATCAGTAGTCTGTACTGCTTATGTTAATTAAATGGTTTCGAAAGAAATGAACTATTGTCAGAAATATATAATCAA 880

Ren gene stop codon

ACGAAGATTAATACATAACATTAATACAAGTTGAACTGAATATGGGCCGACTGTTGTACAGATAACATATATCTATCAAA 960

TCGGCCCACGAAATGTTTTACAGCGCGTACTACAGCATTAATAGAAATTACACCGAAATCATACAAATACTGTAAAATAC 1040

GATTGCGAAATACATGTAGAAAACCACTAGATGATGGCCGTAGAGTTGTCCATATGGTGAAAGTAAGCCAGCATTTATGT 1120

ATCTGTAGAGCACGTCGAAGATTGTAGTTGAACCGTATTTCAACGGTTATACGGTCCCAGTTTTCCCCGCACGGTCTGTC 1200

Potential PolyA signal

CAAATGATCCTTTATACGAAAATACAATGGGTTGGATAATTCCCAGTAAAATACACCCCGGTTTGCCTGTTCAACTGTGA 1280

Ren gene start codon | Rep gene stop codon

TGTCATCACCGGTTCTGTAATCCATTTCAGATGCCGGAATTTGTTGAAGCTGAATTTGTACAAGGCTTAACTTGCAGAAC 1360

TAGGACCTATGCTGTCCTCGGTCGACTCAGGCGTTTGGTACAGAGGGCAATATATGAAATCAAATACCGCATTCTTCAAA 1440

GTCCATTCACGTAAACCAAACTGGTCGGGTCTATCAAGATAATCTTTGTAGCTGCAGTCATTACCAGGATTACACAGAAC 1520

TATTGACGGTACTTCTCCCTTGATCCTGACAGGCTTGCCATATTTGCAATTAGTTAGCCAATGCCGTTGAGACCCGATCA 1600

AATGTTTCCAATGCTTCATACGGAGATAATTTGGCTCGATGTCGTCAATTACATTGTAAAATACATCATTGTTGAATACC 1680

TTCGGATTGAAGTCCAGATGACCACTTATGTAGTTATGGGGGCCCAAAGAACGAGCCCATGCTGTTTTTCCAATCCGACT 1760

GGGCCCTTCAAGAATCAAACTCGTTGGCCGGTCCAAACCAACCTTTGGGACTATTATATTATCTCCATTAGGCCGCGCAG 1840

CGTCATTGATCTGAAAATTATGATTAATCCAATCATTCATCACAGGAGTTATCACAAACGTAGAATAGTTCCAATTAGAT 1920

ATATATTTTTCAGGAGGCTTTGTAAATATCCTGTCATAGTTAGCCTGTAGATTATGATATTGTAATGTAAAAGCTCTGGG 2000

ATCCCTCTCTCTAATTAATTGCAAAGCATCTTCAGCAGAACCACAATTTAAAGCATCTGCATATACGGTTGATAGACTAC 2080

GTCTTCCATCCCTTGTAGATCTACCATCAACCTGGAACTCTCCCCAGTCCATGTAATCCCCTCCTTTCTCGATGTATTCC 2160

A-T Rich Region

TTAACGTCGGAGCTGGATTTAGCTGACTGAATATTTGGATGGAATTTTATAGAAGATGAAGGTGAGTGAAGATCGAATAA 2240

CCTGTTATTTGTTACCTGTGCTCTGCCCTCCAACTGTATTAATACATGTAGATGTGGAGAACCATCCTCATGTAACTCTC 2320

TAGCAACCCGTATGTACTTCTTGTTGCTTGCGAGGGACACTTCTAGCAATTGCTGTAATGCCTCCTCCTTTGTCAGAGAA 2400

Rep gene start codon

CACCTGGCGTAGGTCAGGAAGTAATTCTTAGCATTTAATCTGAACTGCCTCGGTCTGACAGGCATATTTGCTAAATAATA 2480

Potential iteron sequences (type B) [5]

TAGAACACTAAGCTAATGGACCGGCAGTAATGTAAATGAGTCTTACACACTTCTGCAACAGGGAAATTTCAAAAACCCCT 2560

Potential complementary- and virion-sense gene TATA box

Inverted repeat sequence [2]

TAGCAATCGGTGTATGTGTGTATGTTATATAGGCGACCCCCAGAAGGCTCAGAAGGCTCAGAAGGCCCACAAGGCCATCC 2640

GT 2642

A- EGV1 annotated sequence from *D. nummularia* acc. 313

Virion strand origin of replication

Inverted repeat sequence

TAATATTACACGGATGGCCGCCCCGTCCGTACTGCAACGTGTCGAGTTTTGATTGGTCTTCGAAAAAGAAGAAGTACTTG 80

CTTGTCTCCCTTCGTACGTCCACGTGGCTTGTTCTCATTGAGCCATTCAAATTTAAACCGGCGCCCGAATCCTTTTGCCT 160

CTTTCTCAGTTTGTGGGACCCACTAATTTCAAAATAAATATTAAATAAGCAGAAAATAATGGAGTGGGTGCGAGACAATA 240

TTGTTTGTACATAAAGTGGCCCATAGAGGGGAAATAAATTCAAAACCCCTACAGGGCCGTATGATGACCGGTTCAGTTAT 320

TGAGAAACTTGGATTGGAAGAATTAATATTATATAAAGTGCCGAACCGGAGTTACCTCTCAGGCAAGATGGAGAAGGAAG 400

AAATCATGGCCGTATACCTGCTCTTCCTCAGATACGGTCTATCTGCAATAATTAAGAGTGTACATGGTATTTGTATTGCT 480

GAAGCCATCAAGGAAAATCCCTTACCTCTCCTTGAGTATGTCAACTCACTGTAGAAGCACCAAGATGCTATGTACCGAAT 560

Ren gene stop codon

GCTCCGCGAAGCCAGCAAGTCCAGCAAGGATGTACAGGAGGTTACCGATGCATATACAGTGGTCGATAGAGCCAGTAATA 640

AAAAAAAATGAAACTAGAGGACCTCGGTGAAGTTGGGACCTCTAAGGGTGTTGTGTCCACTGTTTGGTGAAGTACATGTA 720

Ren gene start codon

TTTGTATAACCATATATGGAATGAAGTAATGTATCACGTATGTATATTTGTGCTTATTATCAGTTATTCACCAAAATAAA 800

GAATGTCATTTGGGAAAAATTATATGTATGTTAAGACATAACACGGTGCCTTATATAAAAATAAAACACATATTTGCATA 880

ATAAATAATTTTAATACAGGTTGAACTGAACATGGGCCGACTGTTGTACATATAGAATAAACCCATCAAACCGGCCCACG 960

Rep gene stop codon

AAATGAGTAACAACACATATTACAGTATTAATAAAAATTACACCAAAATTATTCAAATACTGCAAGATGCGATTACAAAA 1040

TACATGAAGGAATCCCGCAGATGAAGGCCGTAGATCGAGTTATCCACAGGGTGAAAGATAGCCAGCATTTGTGTATTCGT 1120

AGTGCACGACGTAAATTGTAGTTGAACCATATTTCGACGGTTATACGGTCCCAGTTGTTATTGAACGGTCTGTCTATATG 1200

ATCCTTTATACGGAAATACAATGGCTTGGACAATTCCCAGTAAAATACGCCTCTGTTTGCCTGTACCACAGTAATGTCTT 1280

CACCTGTTCTGAAATCCATCCGAGATACGGTTATGGAATCAAACTCGGCATTCTTCAGAGTCCATTCACGCAAACCTAAG 1360

TTGTCGGGTCGATCAAGATAATCCTTGTAGCTTGAATCATTTCCAGGATTACACAGAACTATAGACGGGACTCCACCCTT 1440

GATCTTGACAGGCTTCCCATACTTGCAATTCGTTTGCCAATCCCGTTGAGACCCAATCAAATGTTTCCAGTGCTTGAGCC 1520

GTAGATAACTTGGTTCTACGTCATCAATGATGTTGTAGAAGATATCATTGTGGAATGTCTTCGGATTGAAGTCCAGATGA 1600

CCACTGATGTAATTATGGGGGCCCAATGACCGAGCCCATGCGGTTTTCCCAACCCGACTGGGCCCTTCAAGGATCAAGCT 1680

GGTGGGCCTGTCCAGCCCAACCTTAGGGATTATTATATTATCTCCATTAGGCCGTGCAGCGTCATTAATATGAAAATTAT 1760

Rep gene start codon

TGTTAATCCAATCATTCATCACAGGATTTACATGAGGATGGTTCTCCACATCTAGATGTGGAGAACCATCCTCACATAAC 1840

TCCCTTGCAACCCGTATGTACTTCTTGTTGCTTGCAAGAGACACCTCCAGTAATTGCCGTAATGCCTCCTACTTGGTTAA 1920

Potential iteron sequences (type B) [5]

AGAACACTTGGCATAAGTCAGGAAGTAATTCTTATCATTCAATCTGAACTGTTTAGGTCTGACAGGCATCTCTGCGAAAA 2000

AAATGGAGAAGAATGGCACTTCTGTAAATAATGGCTTACACACTTCTCCAACAGTATTTTGCAAAAACCCATATCTATGT 2080

Potential complementary- and virion-sense gene TATA box

GTGTAAGTGTGTATCTTATATAGGCGAACCCCCAAAAGCCCCAGAAGGCCATCCGT 2136

Figure S1

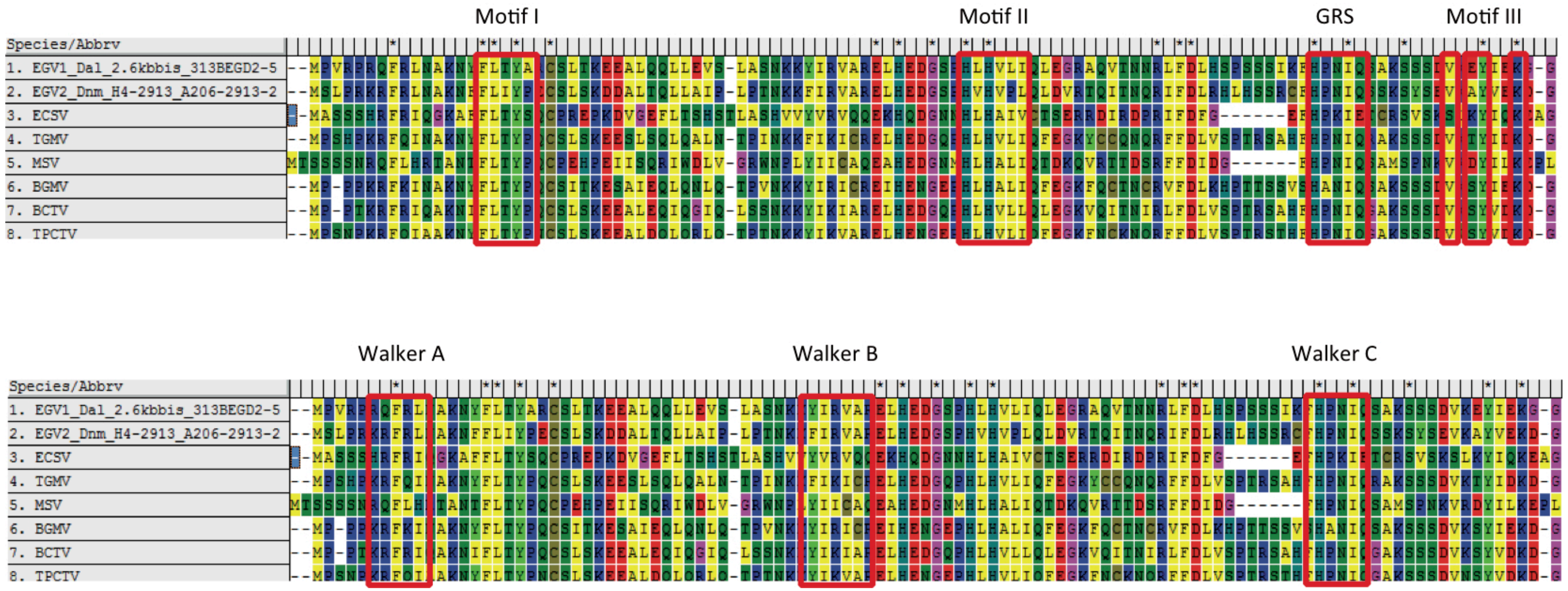
Motif I
Motif II
GRS
Motif III
Species/Abbrv
1. EGV1_Dal_2.6kbbis_313BEGD2-5
2. EGV2_Dnm_H4-2913_A206-2913-2
3. ECSV
4. TGMV
5. MSV
6. BGMV
7. BCTV
8. TPCTV
Walker A
Walker B
Walker C
Species/Abbrv
1. EGV1_Dal_2.6kbbis_313BEGD2-5
2. EGV2_Dnm_H4-2913_A206-2913-2
3. ECSV
4. TGMV
5. MSV
6. BGMV
7. BCTV
8. TPCTV

Figure S2

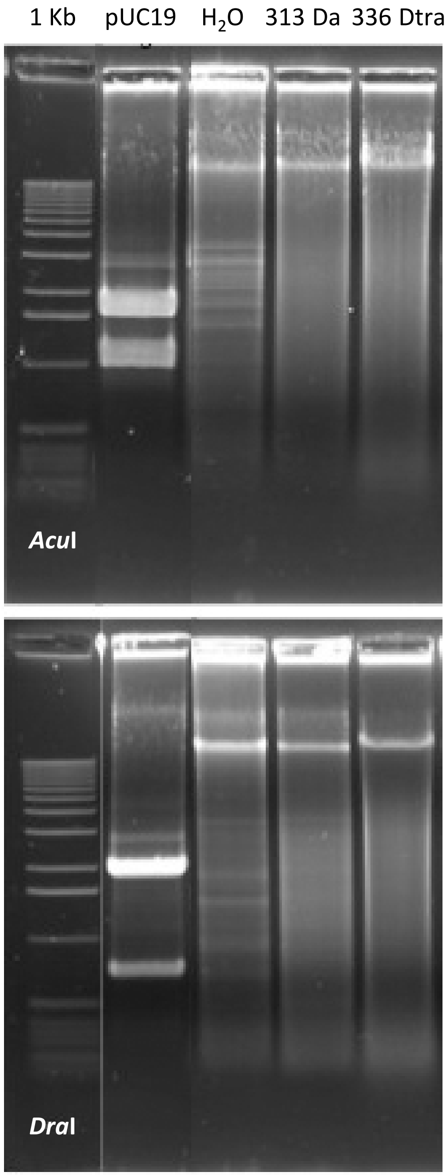

Figure S3

Rolling circle amplification using a cocktail of random and specific primers used for detecting EGV1 or EGV2.

1. Ladder
2. pUC19
3. H_2O
4. *Dioscorea alata* (acc. #313, seedlings)
5. *Dioscorea tranversa* (acc. #336)

Maximum likelihood tree of eighteen 402 bp partial *rep* sequences obtained using the "*ren-ren*" and "*rep-rep*" primer pairs.

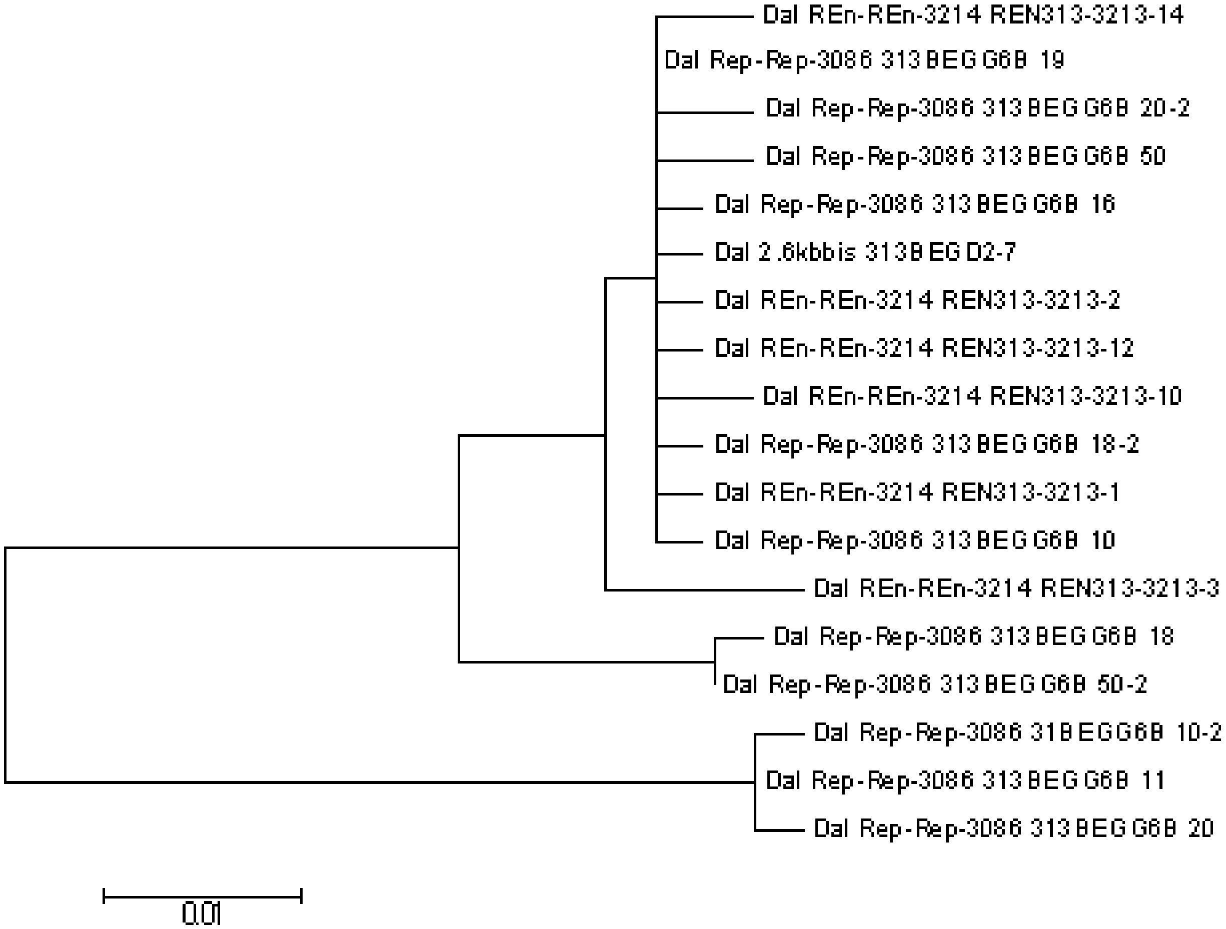

Figure S4

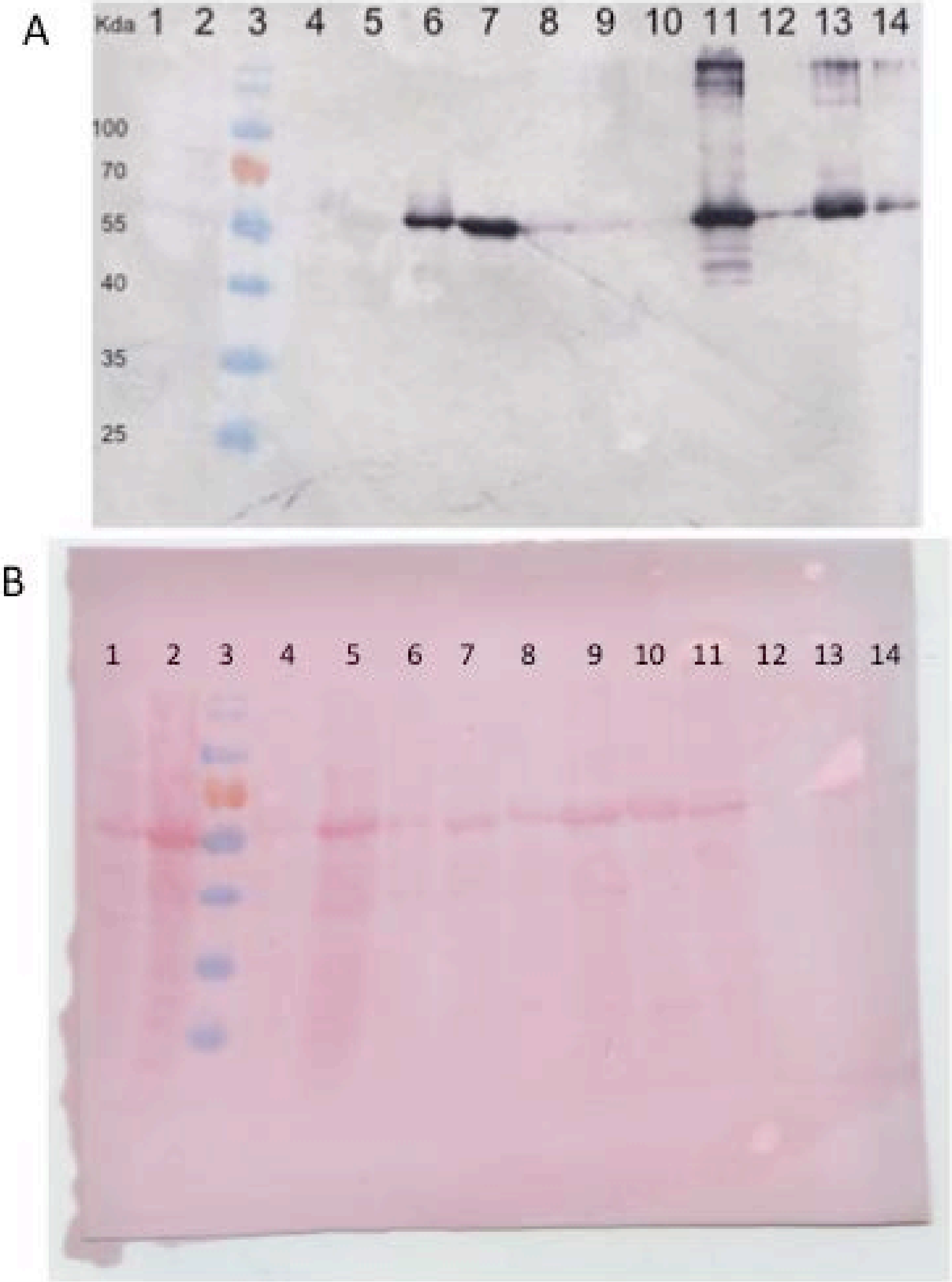

Figure S5

A: Western blot on the total protein extracts of several *Dioscorea* species, using an antibody directed to a Rep peptide of EGV1

B: The nitrocellulose membrane stained with Ponceau S dye for protein detection during western blotting.

Lanes:

1) Proteins extracted from a turnip plant infected by CaMV
2) Tomato plant infected by TYLCV
3) Ladder
4) *Dioscorea trifida* (accession#64)
5) *Dioscorea sansibarensis* (accession#269)
6) *Dioscorea bulbifera* (accession#272)
7) *Dioscorea dumetorum* (accession#47)
8) *Dioscorea togoensis* (accession#114, seedling)
9) *Dioscorea praehensilis* (accession#255)
10) *Dioscorea rotundata* (accession#118, seedling)
11) *Dioscorea nummularia* (accession#335)
12) *Dioscorea alata* (accession#297, seedling)
13) *Dioscorea alata* (accession#313 seedling)
14) *Dioscorea alata* (accession#402 seedling)..

Figure S6

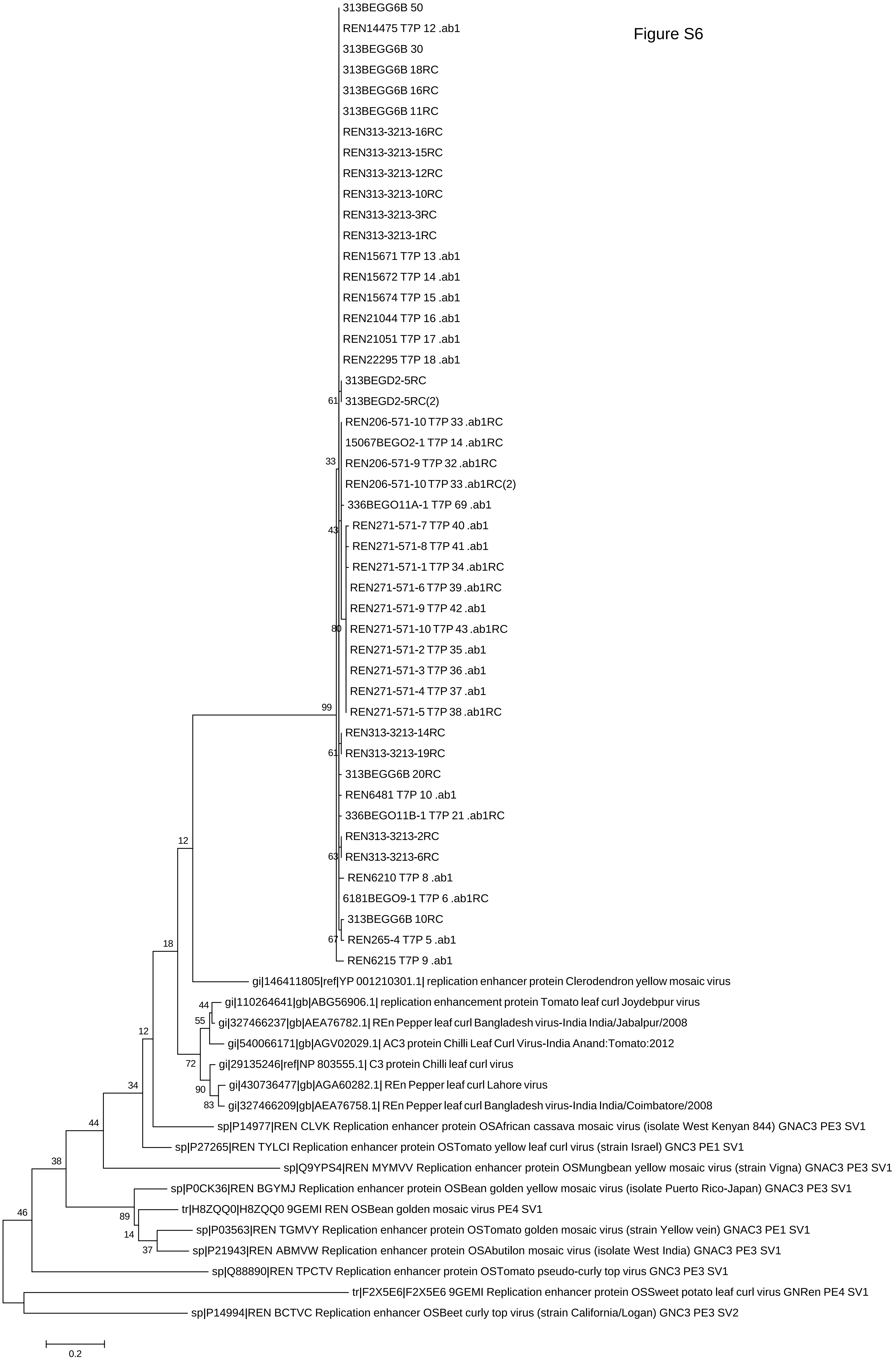
313BEGG6B 50
REN14475 T7P 12 .ab1
313BEGG6B 30
313BEGG6B 18RC
313BEGG6B 16RC
313BEGG6B 11RC
REN313-3213-16RC
REN313-3213-15RC
REN313-3213-12RC
REN313-3213-10RC
REN313-3213-3RC
REN313-3213-1RC
REN15671 T7P 13 .ab1
REN15672 T7P 14 .ab1
REN15674 T7P 15 .ab1
REN21044 T7P 16 .ab1
REN21051 T7P 17 .ab1
REN22295 T7P 18 .ab1
313BEGD2-5RC
61
313BEGD2-5RC(2)
REN206-571-10 T7P 33 .ab1RC
15067BEGO2-1 T7P 14 .ab1RC
33
REN206-571-9 T7P 32 .ab1RC
REN206-571-10 T7P 33 .ab1RC(2)
336BEGO11A-1 T7P 69 .ab1
REN271-571-7 T7P 40 .ab1
43
REN271-571-8 T7P 41 .ab1
REN271-571-1 T7P 34 .ab1RC
REN271-571-6 T7P 39 .ab1RC
REN271-571-9 T7P 42 .ab1
80
REN271-571-10 T7P 43 .ab1RC
REN271-571-2 T7P 35 .ab1
REN271-571-3 T7P 36 .ab1
REN271-571-4 T7P 37 .ab1
99
REN271-571-5 T7P 38 .ab1RC
REN313-3213-14RC
61
REN313-3213-19RC
313BEGG6B 20RC
REN6481 T7P 10 .ab1
336BEGO11B-1 T7P 21 .ab1RC
12
REN313-3213-2RC
63
REN313-3213-6RC
REN6210 T7P 8 .ab1
6181BEGO9-1 T7P 6 .ab1RC
313BEGG6B 10RC
18
67
REN265-4 T7P 5 .ab1
REN6215 T7P 9 .ab1
gi|146411805|ref|YP 001210301.1| replication enhancer protein Clerodendron yellow mosaic virus
44
gi|110264641|gb|ABG56906.1| replication enhancement protein Tomato leaf curl Joydebpur virus
55
gi|327466237|gb|AEA76782.1| REn Pepper leaf curl Bangladesh virus-India India/Jabalpur/2008
12
gi|540066171|gb|AGV02029.1| AC3 protein Chilli Leaf Curl Virus-India Anand:Tomato:2012
72
gi|29135246|ref|NP 803555.1| C3 protein Chilli leaf curl virus
34
90
gi|430736477|gb|AGA60282.1| REn Pepper leaf curl Lahore virus
83
gi|327466209|gb|AEA76758.1| REn Pepper leaf curl Bangladesh virus-India India/Coimbatore/2008
44
sp|P14977|REN CLVK Replication enhancer protein OSAfrican cassava mosaic virus (isolate West Kenyan 844) GNAC3 PE3 SV1
sp|P27265|REN TYLCI Replication enhancer protein OSTomato yellow leaf curl virus (strain Israel) GNC3 PE1 SV1
38
sp|Q9YPS4|REN MYMVV Replication enhancer protein OSMungbean yellow mosaic virus (strain Vigna) GNAC3 PE3 SV1
sp|P0CK36|REN BGYMJ Replication enhancer protein OSBean golden yellow mosaic virus (isolate Puerto Rico-Japan) GNAC3 PE3 SV1
46
89
tr|H8ZQQ0|H8ZQQ0 9GEMI REN OSBean golden mosaic virus PE4 SV1
14
sp|P03563|REN TGMVY Replication enhancer protein OSTomato golden mosaic virus (strain Yellow vein) GNAC3 PE1 SV1
37
sp|P21943|REN ABMVW Replication enhancer protein OSAbutilon mosaic virus (isolate West India) GNAC3 PE3 SV1
sp|Q88890|REN TPCTV Replication enhancer protein OSTomato pseudo-curly top virus GNC3 PE3 SV1
tr|F2X5E6|F2X5E6 9GEMI Replication enhancer protein OSSweet potato leaf curl virus GNRen PE4 SV1
sp|P14994|REN BCTVC Replication enhancer OSBeet curly top virus (strain California/Logan) GNC3 PE3 SV2
0.2

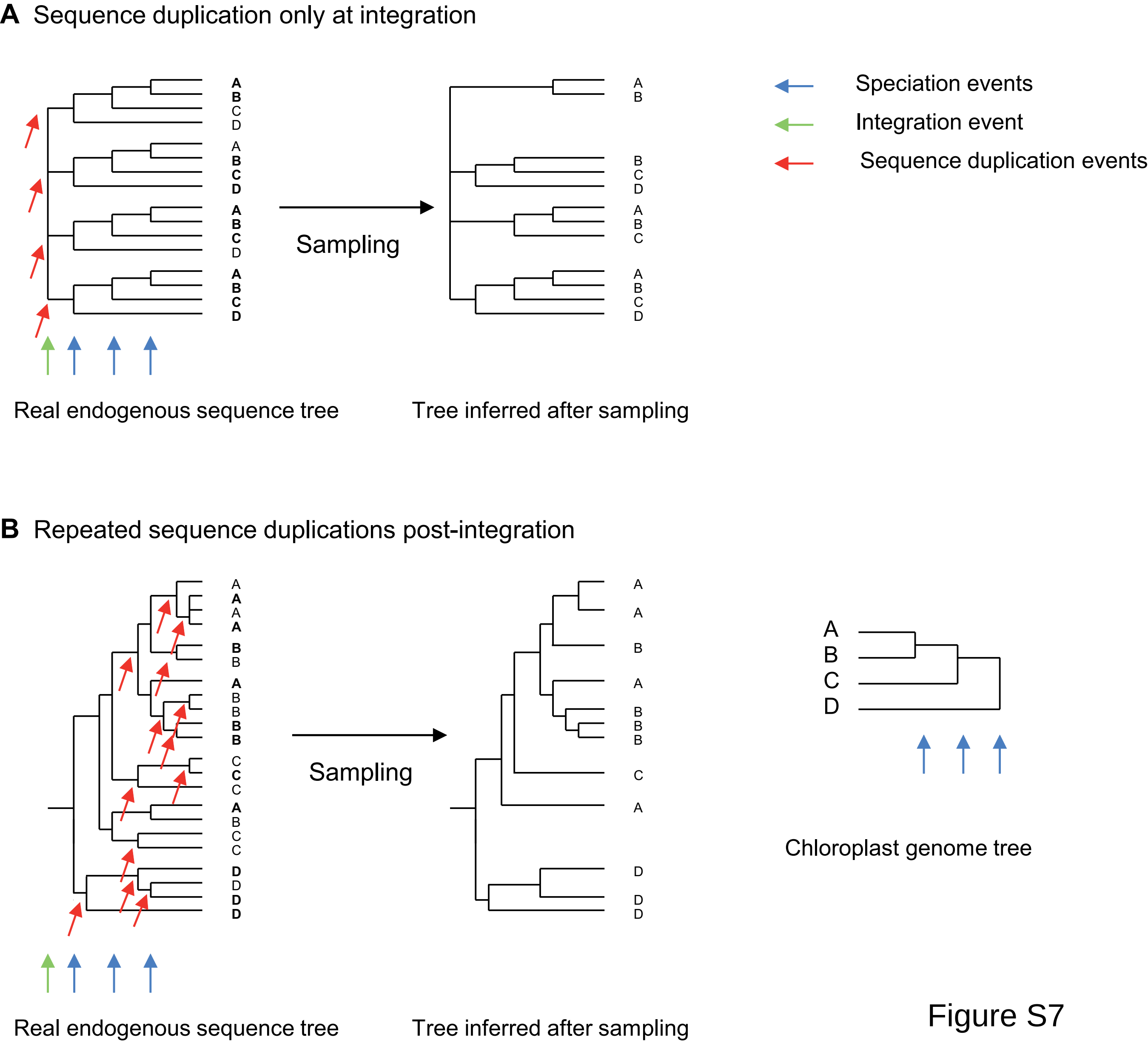
A Sequence duplication only at integration
Speciation events
Integration event
Sequence duplication events
Sampling
Real endogenous sequence tree
Tree inferred after sampling
B Repeated sequence duplications post-integration
Sampling
Chloroplast genome tree
Real endogenous sequence tree
Tree inferred after sampling

Figure S7

Supplementary Table 1. List of primers used in this study

Material and method part	Primer name	Sequence
Full genome amplification of EGV1	YLCV2F	AGATGTGGAGAACCATCCTC
	YLCV1R	GGTTATTCGATCTTCACTCAC
Whole EGV1 and EGV2 *rep* genes amplification	*rep*_EGV1_D_alataF	GCCGGAATTTGTTGAAGCTG
Primer Set1	*rep*_EGV1_D_alataR	GGTCCATTAGCTTAGTGTTCT
Primer Set2	*rep*_EGV1_D_nummulariaF	TCTTCACCTGTTCTGAAATCC
	*rep*_EGV1_D_nummulariaR	GTTGGAGAAGTGTGTAAGCC
Primer Set3	*rep*_EGV2_F	GGAGACGTATACCGAACAAC
	*rep*_EGV2_R	TGGGTTATGAGTTGTTACTTGT
Characterization of multiple EGV1 repeats from *D.alata*	*ren-ren*_D_alataF	GTCATACATCCGGCGTCGT
	*ren-ren*_D_alataR	CAGCTTCAACAAATTCCGGC
	*rep-rep*_D_alataF	AATCCAATCATTCATCACAGGA
	*rep-rep*_D_alataR	GAGGATGGTTCTCCACATCT
Distribution of EGVs within the genomes of *Dioscoreacea* sp.	EGV1_Detection_1F	GTGAGTGAAGATCGAATAACC
	EGV1_Detection_1R	GAGGATGGTTCTCCACATCT
	EGV1_Detection_2F	CGCGCAGCRTCATTRATCTG
	EGV1_Detection_2F	TGGGGWGAGTTYCAGGTTGA
	EGV2_Detection_1F	AGGAATGGAAGTCAAGTCGTA
	EGV2_Detection_1R	AGGTTCAGATTCCAGCTATTC
	EGV2_Detection_2F	TTCCAGGTGTTCTTCTATCTC
	EGV2_Detection_2R	GTCACTACCAASRAAAYGCTTC

Supplementary Table 2. Prevalence of EGV1 and EGV2 sequences among a collection of 9 *D. alata* plants collected worldwide, including two *D. alata* plants grown from true seeds under virus-free conditions.

Species	Accession number	Sampling location	EGV1 292 bp	EGV2 1274 bp
D. alata	301	Brazil	+	+
	440	Costa-Rica	+	+
	71	Cuba	+	+
	408	Ghana	+	+
	314	Haiti	+	+
	313, 402	India (grown from true seeds)	+	+
	MDG009	Madagascar	+	+
	167	Vanuatu	+	+

Supplementary Table 3. Viral sequences recovered from partially purified virions and EST screening.

Procedure/plant sample	Virus-like sequence		Best hit obtained from NCBI Blast (BlastN or BlastX)							
**Plant *vs* EST**	**Name**	**Length (bp)**	**Family**	**Genus**	**Accession Number**	**Species**	**Identity (%)**	**E-value**	**Gene**	**Blast**
**Virion** - *D. alata* - VU564a	EGV1	209	*Geminiviridae*	*Begomovirus*	DQ665866	SiGMBV	71	3e-08	Rep	N
**ESTs** - *D. alata*	HO836974	274	*Caulimoviridae*	*Badnavirus*	L14546	CSSV	72	2e-11	Polyprotein	N
(dbEST Id : 71472229)	HO838291 (EGV2)	316	*Geminiviridae*	*Begomovirus*	HM585443	SiMBoV2	72	1e-20	Rep	N
	Contig3987	2306	*Potyviridae*	*Macluravirus*	AB044386	ChYNMV	72	1e-75	CP	N
	HO833648	325	*Secoviridae*	Unassigned	NP733954	SMoV	45	1e-17	RdRP	X
	Contig1995	2308	*Secoviridae*	*Sadwavirus*	NP620567	SDV	21	3e-6	Polyprotein	X
	Contig2044	2535	*Secoviridae*	Unassigned	NP733984	SMoV	22	1e-16	CP	X
	Contig2047	2027	*Secoviridae*	*Sadwavirus*	NP620567	SDV	23	5e-16	Polyprotein	X
	Contig2049	2577	*Secoviridae*	*Sadwavirus*	BAD12076	SDV	21	5e-19	CP	X
	Contig2216	2950	*Secoviridae*	Unassigned	EU419645	BRNV	84	3e-08	RdRP	N

Supplementary Table 4. Selection analyses results (*N. benthamiana*). For each test, each clade was assigned to a foreground partition (FG), or one of two background partitions (BG1 and BG2). We report p-values for significance tests for purifying selection along foreground lineages, and the parameter point-estimates for the foreground partition under the alternative model (which allows purifying selection).

GRD3	GRD5	Connecting branch	p-value for neg. selection	ω_1	p_1
BG 1	BG 2	**FG**	**7.9×10^{-12}**	0.043	1
BG 1	**FG**	BG 2	0.44	0.75	1
**FG**	BG 1	BG 2	0.995	0.94	0.65
**FG**	**FG**	BG	0.55	0.82	1
